# European agroforestry has no unequivocal effect on biodiversity: a time-cumulative meta-analysis

**DOI:** 10.1186/s12862-021-01911-9

**Published:** 2021-10-23

**Authors:** Anne-Christine Mupepele, Matteo Keller, Carsten F. Dormann

**Affiliations:** 1 Nature Conservation and Landscape Ecology, University of Freiburg, Tennenbacherstr. 4, 79106 Freiburg, Germany; 2 Biometry and Environmental System Analysis, University of Freiburg, Tennenbacherstr. 4, 79106 Freiburg, Germany

**Keywords:** Species richness, Silvopasture, Silvopastoral, Silvoarable, Arthropods, Birds

## Abstract

**Background:**

Agroforestry is a production system combining trees with crops or livestock. It has the potential to increase biodiversity in relation to single-use systems, such as pastures or cropland, by providing a higher habitat heterogeneity. In a literature review and subsequent meta-analysis, we investigated the relationship between biodiversity and agroforestry and critically appraised the underlying evidence of the results.

**Results:**

Overall, there was no benefit of agroforestry to biodiversity. A time-cumulative meta-analysis demonstrated the robustness of this result between 1991 and 2019. In a more nuanced view silvopastoral systems were not more diverse in relation to forests, pastures or abandoned silvopastures. However, silvoarable systems increased biodiversity compared to cropland by 60%. A subgroup analysis showed that bird and arthropod diversity increased in agroforestry systems, while bats, plants and fungi did not.

**Conclusion:**

Agroforestry increases biodiversity only in silvoarable systems in relation to cropland. But even this result is of small magnitude, and single-study effect sizes were heterogeneous with sometimes opposing conclusions. The heterogeneity suggests the importance of other, usually unmeasured variables, such as landscape parameters or land-use history, influencing biodiversity in agroforestry systems.

**Supplementary Information:**

The online version contains supplementary material available at 10.1186/s12862-021-01911-9.

## Background

Agroforestry is a collective name for diverse land-use systems integrating tree husbandry with livestock or arable cultivation [1, 2]. It is a historical element of the European landscape with traditional agroforestry approaches such as large fruit orchards with extensive livestock grazing. Nowadays, new forms of agroforestry, e.g. short-rotation coppice in combination with crop rows, are implemented in some places [3]. Agroforestry is subdivided into silvopastoral systems, grazed by livestock or used for fodder production, and silvoarable systems, in which crops are grown among trees [4]. Fields where trees are grown only at the edge, such as stream-side management zones or hedgerows adjacent to arable land, are also occasionally subsumed under agroforestry systems [4]. In these cases, the herbaceous and wooded components are usually not managed together and may have different owners. In our study, trees or shrubs adjacent to fields or pastures are not considered.

Biodiversity is threatened and particularly steep declines have been observed in intensively used agricultural areas [5–8]. Compared to monocultures, agroforestry systems increase heterogeneity in the landscape structure and potentially lead to increased biodiversity [5, 9–12]. Demonstrating a clear benefit for biodiversity could favour future subsidies for agroforestry systems by the Common Agricultural Policy or its successor policies [13–17].

The benefits for biodiversity in agroforestry systems have been investigated particularly in the tropics, indicating that biodiversity can be improved by agroforestry in degraded and intensively cropped areas, while it remained lower in comparison to primary and secondary forests [18–20]. In the temperate zones, studies for species groups such as birds [21] and invertebrates [22] have shown equivocal effects on biodiversity. An earlier meta-analysis found a net increase of biodiversity across taxa and agroforestry systems in Europe; however it did not provide detailed information on the heterogeneity and robustness of their findings and was based on a broader definition of agroforestry including field-adjacent hedgerows and riparian buffers [23]. Here we provide an evidence update and a more explicit discussion of biodiversity in direct comparison to forests and agriculture and assess the robustness of the results by answering the following research questions:

1. What is the effect of agroforestry on biodiversity relative to forests, pastures, cropland or abandoned, shrub-encroached agroforestry?
2. Is the effect of agroforestry on biodiversity influenced by environmental variables, specifically the kind of agroforestry system (silvopasture or silvoarable), sampling method, the specific measure of biodiversity, sampling year, country, climate and the reference used?
3. How strong and robust is the underlying evidence of these results?

## Results

**Fig. 1:**
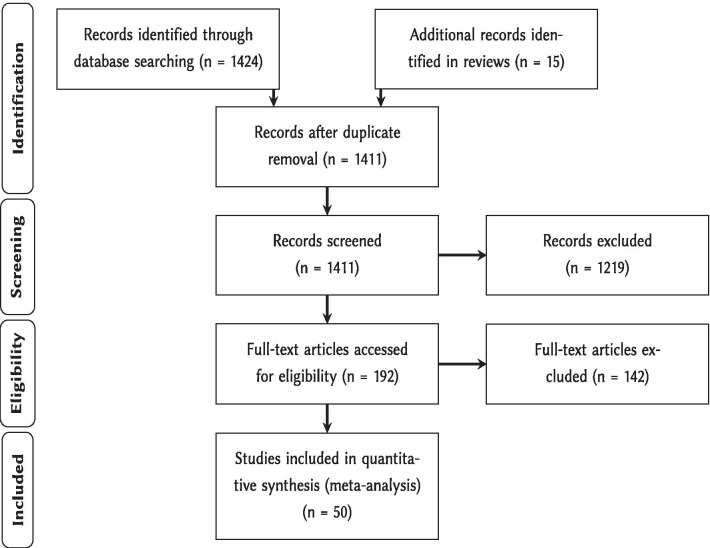
PRISMA flow diagram [24, 25]

The literature search resulted in 1411 records from which 50 articles met all inclusion criteria (Fig. 1, Additional file 1, 2 and 7). Unique combinations of agroforestry systems (silvoarable or silvopastoral), control types (forest, cropland, pasture or abandoned agroforestry systems) and taxonomic groups per study led to 69 effect sizes used in the meta-analysis.

**Fig. 2:**
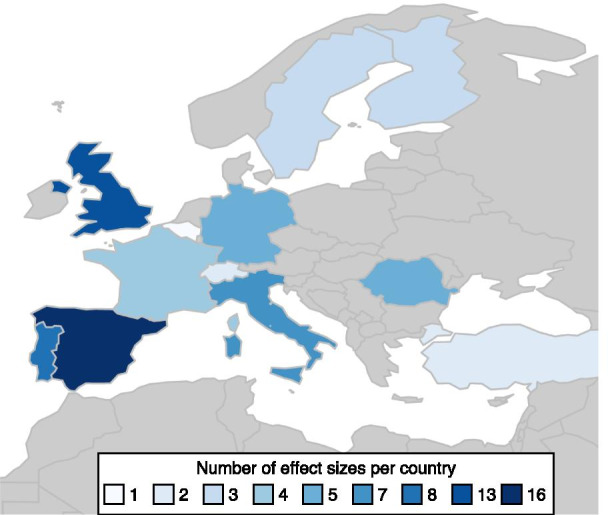
Map of Europe with the number of effect sizes per country

Studies had been conducted in sites all across Europe and covered data from 1984 to 2019 (Fig. 2). The majority of study sites were located in the Mediterranean with 12 studies from Spain, 8 from Portugal, 5 from Italy and one each from France and Turkey. There were fewer studies from the temperate central European climate. They ranged from the United Kingdom (6), Romania (4), France (2), Germany (2), Switzerland (2) and Belgium (1) to northern Italy (1). The boreal region was represented by four studies from Sweden and two from Finland.

Agroforestry systems were predominately silvopastoral (36 studies, 52 effect sizes), while silvoarable systems were less often a topic of research (13 studies, 17 effect sizes). The impact of agroforestry on biodiversity was evaluated by comparing agroforestry systems to a control type. Most often this control type was a pasture (23 effect sizes), followed by forests (21 effect sizes), abandoned agroforestry systems (13 effect sizes) and cropland (12 effect sizes).

**Fig. 3:**
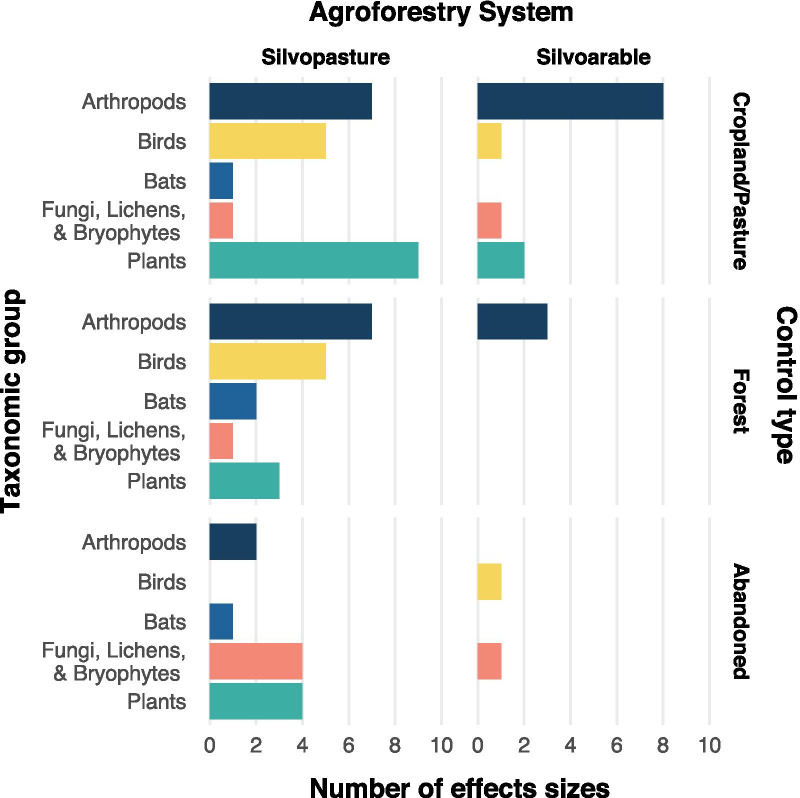
Number of effect sizes in each combination of agroforestry system, comparator and biodiversity group

Biodiversity was measured in different taxonomic groups and reported at various levels of detail across studies (Fig. 3). Some studies for example lumped all arthropods, whereas others reported diversity of carabid beetles only. We clustered biodiversity measures into five groups: arthropods, birds, bats, plants and one group with fungi, lichens and bryophytes. Biodiversity effects were mainly measured based on differences in species richness. Five studies with seven effect sizes used other measures, namely family richness [26, 27], log-series [28, 29] or Shannon index [30].

### Effects of agroforestry on biodiversity

**Fig. 4:**
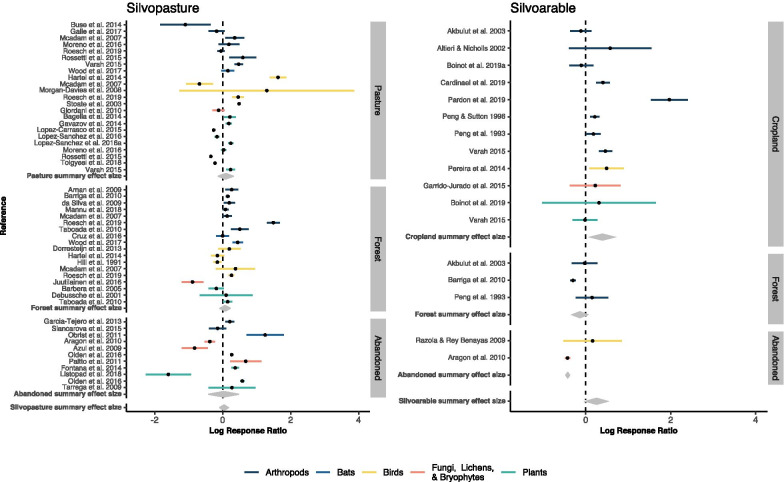
Forest plot for silvopasture and silvoarable systems with subgroup summary effect sizes (grey diamonds) per system (silvoarable, silvopastoral) and per control type (pasture/cropland, forest or abandoned agroforestry system)

The results of the meta-analysis show that there is no general benefit of agroforestry systems to biodiversity (summary effect size $= 0.1, 95\%$ CI $= [-0.03, 0.23], \,z_{df=68}=1.47,\, p=0.14$, Additional file 5). The studies’ individual effects sizes show substantial between-study variability (Q $= 6229,\, p<0.0001;\, I^2 =98.9\%$; Fig. 4). Some of this heterogeneity was attributed to systematic differences in environmental variables, and ‘taxonomic group’, ‘control type’ and ‘agroforestry type’ could explain 13.5% of the heterogeneity (marginal $R^2$).

A subgroup analysis for each agroforestry system, further distinguishing biodiversity effects depending on the control type, revealed that silvoarable systems were significantly more diverse than cropland (Fig. 4 right plot, ‘Cropland’ summary effect size $$= 0.46, 95\%$$CI $$ = [0.1, 0.82], z_{df=11}=2.52, p=0.012$$), with 1.6 times more species in the agroforestry system than in cropland. Comparing the biodiversity of silvoarable systems to forests, they did not differ significantly, but showed a tendency towards higher diversity in forests. In silvopastoral systems, none of the subgroup effect sizes was significant (Fig. 4 left plot). Effect sizes were very heterogeneous and with partly opposing effects, such as forests harbouring a higher bird diversity in relation to agroforestry in one study [31, moderate-evidence study] and the other way around in another study [32, moderate-evidence study].

**Fig. 5:**
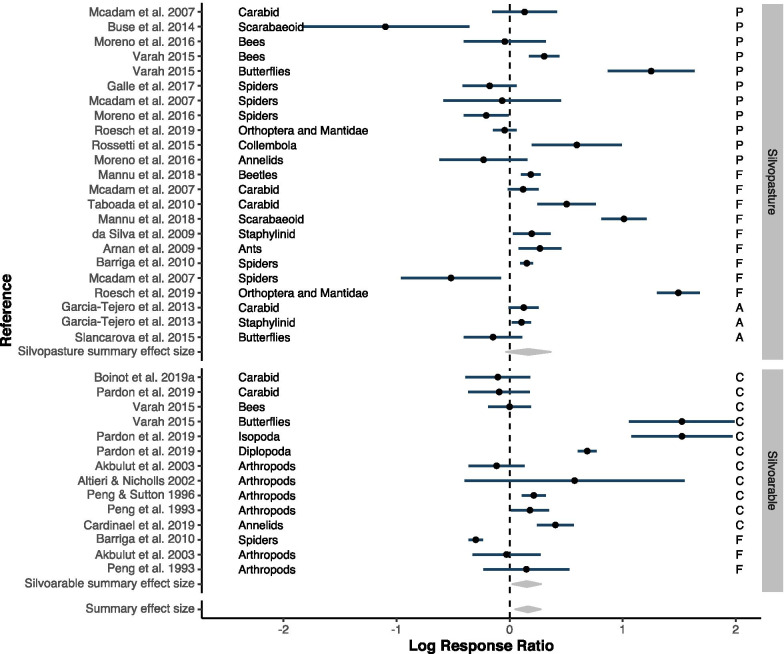
Arthropod subgroup analysis with summary effect sizes (grey diamonds). Taxonomic groups are provided in more detail depending on reported groups in primary studies. Letters on the right side reflect the first letter of the control type (P=Pasture, C=Cropland, F=Forests, A=Abandoned)

A subgroup analysis of taxonomic groups showed that birds and arthropods are significantly more diverse across all agroforestry systems (bird summary effect size $$= 0.23, 95\%$$CI $$ = [0.012, 0.44], z_{df=11} = 2.07, p = 0.038$$; arthropods summary effect size $$= 0.3, 95\%$$CI $$ = [0.016, 0.59], z_{df=26} = 2.07, p = 0.038$$). For arthropods a higher resolution was available with subgroups on different taxonomic levels, such as bees or spiders. This increased the number of effect sizes from 27 to 41 as the number of unique combination of taxonomic group and agroforestry system increased. None of the most replicated groups, i.e. beetles, bees and spiders, showed a consistent diversity response to agroforestry (Fig. 5).

### Sensitivity analysis and the underlying evidence

The quality of studies included in this meta-analysis ranged from weak to strong evidence [compare with 33]. Some studies were based on a replicated and controlled design providing the strongest evidence, whereas others used before-after comparison or an observational gradient. We adjusted the study weights according to their level of evidence to assign a lower weight to weaker studies (Additional file 3). The results of the evidence-weighted meta-analysis did not lead to different conclusions and confirmed the results of the traditional inverse-variance-weighted meta-analysis (level-of-evidence-weighted summary effect size $$= 0.093, 95\%$$CI $$ = [-0.003, 0.19]$$).

Beside the weighting of studies, missing studies due to a publication bias is another obstacle for robust meta-analytical results. According to the funnel plot and Egger’s regression test, no publication bias is detectable in our data (Additional files 5 and 6, intercept of Egger’s regression $$ = 0.77, t = 0.03, p = 0.98$$).

**Fig. 6:**
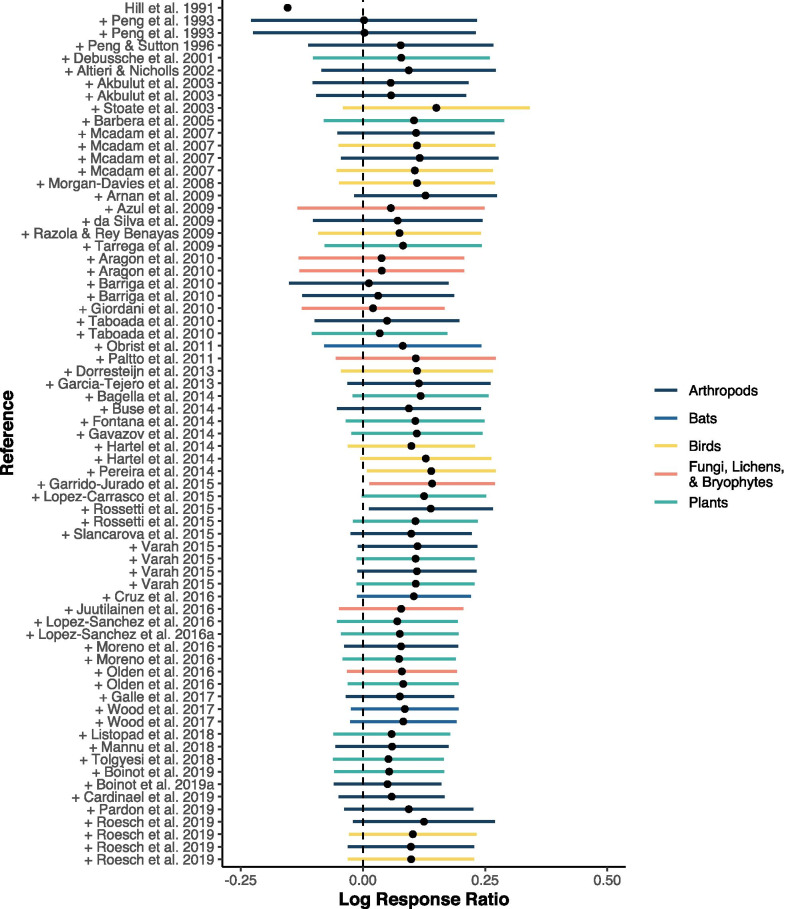
Cumulative forest plot, showing the summary effect sizes with always one individual effect size added over time. Colour code for the biodiversity groups indicate no clustering of any group in the time series

Given that an earlier meta-analysis [23] has found a significant effect of agroforestry on biodiversity, we were interested in the change of the conclusion over time. A cumulative meta-analysis shows that there is a tendency of evidence for a beneficial effect of agroforestry over time. But only at one point in time, in early 2015, when the studies from Garrido-Jurado et al. [34] and Rossetti et al. [35] were added, the confidence interval was above zero (Fig. 6). A meta-analysis conducted in early 2015 would have resulted in an overall significant positive effect of agroforestry on biodiversity. At all other times, between 1991 and today, there is no general evidence for a beneficial effect of agroforestry on biodiversity, and the conclusion remains robust over the time. Another possible bias could have been introduced by systematically investigating a particular taxonomic group during a certain time period, e.g. a peak of bird studies in the 1990s. Taxonomic groups, however, ranged across the whole time period and did not cluster and as such did not bias the results (Fig. 6, colour code).

## Discussion

European silvoarable systems host higher biodiversity than cropland, but show a tendency towards lower diversity than forests. In silvopastoral systems there was no evident benefit over either single-use system. Abandoning traditional agroforestry systems and leaving them to shrub encroachment and natural succession did not increase or reduce biodiversity systematically, as was suggested in other studies, and is likely to depend on the number of years since abandonment [36, 37]. Birds and arthropods exhibited significantly higher diversity in agroforestry systems in our subgroup analysis. The higher diversity of arthropods in agroforestry could not be traced back to any particular subgroup such as beetles, spiders or bees. Even within the taxonomic subgroups effects were heterogeneous. Spider diversity, for example, was found to be higher in agroforestry compared to a forest in one study [38, moderate-evidence study], but showed the opposite effect in another study [39, weak-evidence study].

Agroforestry covers around 10% of the agricultural area in the European Union [15]. Among them are traditional and very long established agroforestry sites, such as the Mediterranean Dehesas and Montado, traditional Spanish and Portugese silvopastures [3]. Land-use history, i.e. the age of the agroforestry system and the previous land-use type, may have a strong impact which is hardly reported or even known to the primary-study authors [20]. As such an older agroforestry system may harbour a different biodiversity than a newly established one; and the same holds for an old-grown forest relative to a more intensively managed younger forest site.

Additional unmeasured drivers operating at the landscape scale may equally determine the biodiversity. The implementation of agroforestry at the field scale does not guarantee the viability of populations of tree-dependent species, but could host these species if additional forest patches are found nearby [40, 41]. Invertebrates for example profit from a diverse landscape beyond the field scale [42, 43]. Our conclusion are largely based on species richness comparison; communities may well differ in their composition beyond richness [compare e.g. 22, 44, 45]. For conservation decisions, variables such as the occurence of rare and endangered species may be additionally relevant.

### Robustness of meta-analytical results

Meta-analysis of systematically searched literature provides evidence that is stronger than individual studies, unsystematic literature searches and qualitative synthesis [28, 46, 47]. Conclusions drawn from a meta-analysis nevertheless depend on the robustness of the result, i.e. whether minor changes such as alternating the weighting could reverse the conclusion. Weighting of studies traditionally occurs by inverse variance without considering the differences in study quality and design. In previous work, the underlying evidence and thus the reliability of individual study results was shown to be distinct depending on their study design [48, 49]. Weighting studies proportional to the evidence underlying each individual study is an alternative to the traditional weighting. In our case, results did not change with the alternative weighting approach, but can confirm the robustness of our conclusions.

Meta-analysis has established in ecology and as such updates of already existing meta-analyses can show how and whether conclusions may change over time. In a cumulative meta-analysis, adding new studies according to their publication date, we did not observe a declining effect as observed in other meta-analyses, but the effect remained stable despite very heterogeneous individual study results [50]. In our study we also found that other environmental variables have an influence on the agroforestry-biodiversity relationship. Meta-analysis builds on what is found in the literature, and additional categorical environmental variables used as moderators in meta-analytical models are rarely balanced. The results of our analysis is robust over time and adding new studies is unlikely to impact the results [51], but systematically adding studies on silvoarable systems, which in the current meta-analysis make up only one third of silvopastoral-study contribution, could well influence the results. An increasing number of silvoarable studies may move the overall effect size further towards the positive end and eventually turn the combined result to be significantly positive. Given that silvopastoral systems are dominant in Europe, we are nevertheless convinced that the ratio of silvopastoral and silvoarable studies in our meta-analysis reflects the proportion in which agroforestry systems in Europe occur and provide representative results [15].

Reproducibility of results is a sign of robustness, but challenging and often frail [52, 53]. The present meta-analysis and the analysis from Torralba et al. [23] have resulted in different conclusions, as we failed to reproduce their results. While Torralba et al. [23] concluded that agroforestry has a positive effect on biodiversity in general, we could confirm a benefit only in relation to cropland. A possible explanation is the different set of studies used in their meta-analysis. Their definition of agroforestry includes studies on hedgerows and woody riparian buffers bordering agricultural field, which we did not consider as agroforestry as they are not actually under silvicultural use. They have also missed study results from biodiversity studies that reported disadvantages of agroforestry [e.g. 54, 55]. Successfully consolidating different results could be achieved by clearly communicating the context in which they apply, providing code and data used in the analysis to posthoc identify differences, and a ranking scale communicating, how confident scientists are with their statements. This is desirable to support decision makers, and has been demonstrated for the policy-relevant IPCC reports [56–58]. In this specific case, where reviews with the same attempt on similar data yield different results, such a confidence statement may indicate that both reviews are indeed very similar in their assessment. In a subgroup analysis of Torralba et al. [23], distinguishing between fungi, arthropods, plants and birds, only birds were significantly positive, which we could confirm in our analysis. In contrast to their results, we have to emphasize that results are heterogeneous. Our review suggests weak effects, and we are only moderately confident about these findings, supposing that the main driver for biodiversity cannot be found in agroforestry but may lie at the landscape scale or be dependent on land-use history.

## Conclusion

Agroforestry increases biodiversity in silvoarable systems compared to cropland and in general for birds and arthropods, but benefits were small and there was no overall positive effect of agroforestry on biodiversity. Outcomes were influenced by the heterogeneity of effect sizes and silvopastoral systems did not show a benefit over either single-use system. While previous reviews were enthusiastic and considered agroforestry to have led to an increase in biodiversity [23], we need to call for caution. In the present evidence assessment, we have identified only few studies providing results based on strong evidence, and those paint a heterogeneous picture, suggesting other variables to interact with positive or negative effects from agroforestry. Systematic reviews and meta-analyses are providing the best available evidence, but they do not automatically guarantee reproducibility. They depend on the quality, quantity and comparability of studies used in the analysis. We suggest to resolve these issues by a detailed reporting, data provision and the communication of heterogeneity. Our study provides results embedded in the context in which agroforestry can lead to a benefit for biodiversity. Together with the knowledge available about the impact of agroforestry on carbon sequestration and other ecosystem services, our results can enrich the discussion on how future subsidies from the Common Agricultural Policy of the European Union can further incorporate agroforestry measures. Future studies on landscape parameters and land-use history are required to disentangle the context in which agroforestry is beneficial for biodiversity.

## Methods

We reviewed the literature on biodiversity in European agroforestry systems and synthesized the results in a meta-analysis. This review is based on the standards of the Collaboration for Environmental Evidence [24, 59–61]. It goes beyond these standards by additionally performing a sensitivity analysis with studies weighted by their evidence to identify the robustness of the results [56].

### Literature search

We used search terms and their synonyms related to ‘biodiversity’, ‘agroforestry’ and ‘Europe’ in the Web of Science to identify the relevant literature (Box 1). Reviews revealed by the Web of Science search were scanned for additional references. In the first screening of articles, we sighted title and abstract and excluded publications that did not fulfil the inclusion criteria (Box 2). In a second screening, we read the full text and applied additional inclusion criteria (Box 2). If an article was included, we extracted the mean diversity, standard deviation and sample size in an agroforestry system and its corresponding reference (control site) along with environmental variables (see Table 1 for the full list of environmental variables). Observational studies were included if a good proxy for a control site was available. This was the case for studies about species groups with limited mobility (e.g. plants or Collembola) looking at distance gradients. The values most distant to the agroforestry treatment served as a control in our meta-analysis (see also Additional file 4 column ’comments’ for more details). WebPlotDigitizer was used to extract data points from figures [62]. Unique combinations of agroforestry system, control type and taxonomic group were considered from each article.

**Table 1:** Environmental variables

Variable name	Categories
Agroforestry system	Silvopastoral; silvoarable
Control type	Forest, cropland, pasture or abandoned agroforestry systems (generally described as shrub-encroached)
Sampling methods	Transects with sweep netting; pitfall traps; pan traps; recording and various other methods
Diversity measure	Species richness, family richness or Shannon diversity
Sampling year	Numeric ranging from 1984 to 2019
Country of study location	European country
Climate zone	Mediterranean (including two summer-moist Atlantic studies), temperate Central European or boreal

Box 1: Search string used in the Web of Science initially in January 2016 with updates in 2018, 2019 and last on 14th February 2020. The search covered the following databases: Web of Science Core Collection, BIOSIS Citation Index, BIOSIS Previews, Current Contents Connect, Data Citation Index, Derwent Innovations Index, KCI-Korean Journal Database, MEDLINE, SciELO Citation Index, Zoological Record. Search options in the Web of Sciences were set to ‘all years’ and ‘all languages’ Topic: (*diversity OR "species richness" OR "species composition") AND: (Agroforest* OR agro-forest* OR silvopast* OR *silvoarabl* OR dehesa OR "alley* cropping" OR "wood* pasture*" OR "forest* farming*" OR "orchard* intercropping" OR "scatter* tree*" OR "grazed orchard" OR montado) AND: (Europe OR Albania OR Andorra OR Armenia OR Austria OR Azerbaijan OR Belarus OR Belgium OR "Bosnia and Herzegovina" OR Bulgaria OR Croatia OR Cyprus OR "Czech Republic" OR Denmark OR Estonia OR Finland OR France OR Georgia OR Germany OR Greece OR Hungary OR Iceland OR Ireland OR Italy OR Latvia OR Liechtenstein OR Lithuania OR Luxembourg OR Macedonia OR Malta OR Moldova OR Monaco OR Montenegro OR Netherlands OR Norway OR Poland OR Portugal OR Romania OR Russia OR Serbia OR Slovakia OR Slovenia OR Spain OR Sweden OR Switzerland OR Ukraine OR "United Kingdom" OR England OR Wales OR Scotland) .

Box 2. Inclusion criteria for studies to be included in this review Inclusion criteria for title and abstract screening

- Study sites are located in Europe. Europe ranges from Iceland to the Ural Mountains and from Norway to the Mediterranean Sea and the Black Sea.
- The study is done in an agroforestry system, whereas agroforestry is defined by an area covered by crops or livestock and trees in an alternating way. Buffer strips and hedgerows only bordering an agricultural field or pasture were not considered.
- The study provides information on biodiversity in an agroforestry system.

Additional inclusion criteria for full-text screening

- The study should not discuss conceptual approaches or introduce new methods without quantifying biodiversity in agroforestry.
- If a study reviewed other primary studies, references were verified for inclusion.
- Average species richness or another quantifiable biodiversity measure, such as Shannon diversity, needs to be extractable for an agroforestry system and a corresponding control type in relation to their sample size.
- Full text was searched in the internet open access and through the subscriptions hold by the University of Freiburg. Authors were contacted via e-mail if we could not find or access the full text online. If all these attempts to access the full text of an article failed, the study could not be included.

### Analysis

Meta-analysis is based on effect sizes and here we used log response ratios to compare the biodiversity between an agroforestry site and its corresponding control site [63, 64]. The summary effect of agroforestry on biodiversity was estimated by running a random-effect model, with a random effect for study, and no fixed effect [65]. Heterogeneity was tested with a Q-test for heterogeneity and additionally given by $I^2$, the ratio of heterogeneity (i.e. between-study variability) to the total variability (i.e. sum of between- and within-study variability) [66, 67]. If heterogeneity accounts for large amounts of the total variability, additional environmental variables (moderators), such as sampling method or study location (see Table 1) may improve the model by further explaining parts of the heterogeneity. This was investigated with a mixed-effects model with fixed-effects selection based on a likelihood-ratio test of the maximum-likelihood fits [68]. Marginal $R^2$ was given to identify the amount of heterogeneity that could be explained by the selected fixed effects [69, 70]. If the mixed-effects model identified categorical environmental variables influencing the agroforestry-biodiversity relationship, a subsequent subgroup meta-analysis was performed to identify under which circumstances agroforestry has an impact on biodiversity. Analysis was realized in R 4.0.2 using packages ‘metafor’, ‘nlme’ and ‘MuMIn’ [64, 71, 72], see Additional files 4 and 5 for details and R code].

#### Sensitivity analysis

Studies are traditionally weighted according to their inverse variance. This method has been criticized for being prone to bias especially with small sample sizes [73]. We tested the robustness of the results by adjusting the weighting by the underlying evidence of each study [compare with 33]. For this purpose, the traditional inverse variance weighting was modified by multiplying the weight with the level of evidence each study provided to reduce the influence of low-evidence studies on the summary effect size. The level of evidence was assessed with help of an evidence assessment tool considering the underlying study design (e.g. case-control or observational) and quality criteria, such as sample size [56]. Publication bias, i.e. the tendency of statistically significant results being more often published than non-significant results, was assessed based on a funnel plot and an Egger’s regression test [64, 68, 74].

## Additional file

**Additional file 1.** Literature Search Screening. It contains meta information about the abbreviations and the list of literature that was screened: 1439 rows - one row per study - and 14 columns containing information on author, title and journal as well as the inclusion and exclusion criteria.

**Additional file 2.** Literature included in meta analysis. It contains the full reference of all the 50 studies retained after the literature search (Additional file 1) and included in the meta analysis. It is also available in the bib format (.bib) for the integration in a reference software.

**Additional file 3.** Level of evidence assessment. Every column refers to one study. The spreadsheet template is taken from the supplementary material of [28].

**Additional file 4.** Data for the meta-analysis on biodiversity. The file is a semicolon separated table (.csv) that can be opened in a spreadsheet. It has 29 rows and 21 columns containing information required for the meta-analysis. Meta information about the abbreviations used is found in Additional file 5.

**Additional file 5.** Description of data tables and R-Code of the analysis. The file is in the R-Markdown format (.rmd) and can be opened via R-Studio or any other text editor. The Appendix 5 is also provided in the html format including the R-Output of the analysis.

**Additional file 6.** Funnel Plot. The file is a portal document format (.pdf) with the funnel plot of the analysis.

**Additional file 7.** Prisma checklist. It is a checklist according to the Preferred Reporting Items for Systematic Reviews and Meta-Analyses (PRISMA) guidelines (http://www.prisma-statement.org/).

## Data Availability

All data required for the replication of the analysis are available in the supplementary material.
