# Supplementary material for: European agroforestry has no unequivocal effect on biodiversity: a time-cumulative meta-analysis
Source: BMC Ecol Evol. 2021 Oct 23;21:193. doi: 10.1186/s12862-021-01911-9 (PMC8541809; doi:10.1186/s12862-021-01911-9)
Supplement: Supplementary file 2 — Additional file 2. Literature included in meta analysis. It contains the full reference of all the 50 studies retained after the literature search (Additional file 1) and included in the meta analysis. It is also available in the bib format (.bib) for the integration in a reference software. [file 12862_2021_1911_MOESM2_ESM.zip › Appendix2_References_of_included_studies.pdf]

European agroforestry has no unequivocal effect on biodiversity: a time-cumulative meta-analysis Additional file 2: Reference list of studies included in the meta-analysis

Anne-Christine Mupepele, Matteo Keller, Amelie Göbel, Carsten F. Dormann

The following 50 studies were included in our meta-analysis.

References

Akbulut S, Keten A, and Stamps WT. 2003. Effect of alley cropping on crops and arthropod diversity in Duzce, Turkey. *Journal of Agronomy and Crop Science* **189**: 261–269.

Altieri MA and Nicholls CI. 2002. The simplification of traditional vineyard based agroforests in northwestern Portugal: Some ecological implications. *Agroforestry Systems* **56**: 185–191.

Aragón G, López R, and Martínez I. 2010. Effects of Mediterranean dehesa management on epiphytic lichens. *Science of the Total Environment* **409**: 116–122.

Arnan X, Gracia M, Comas L, and Retana J. 2009. Forest management conditioning ground ant community structure and composition in temperate conifer forests in the Pyrenees Mountains. *Forest Ecology and Management* **258**: 51–59.

Azul AM, Castro P, Sousa JP, and Freitas H. 2009. Diversity and fruiting patterns of ectomycorrhizal and saprobic fungi as indicators of land-use severity in managed woodlands dominated by Quercus suber- a case study from southern Portugal Anabela. *Canadian Journal of Forest Research* **39**: 2404–2417.

Bagella S, Filigheddu R, Carmela M, *et al.* 2014. Contrasting land uses in Mediterranean agro-silvo-pastoral systems generated patchy diversity patterns of vascular

plants and below-ground microorganisms. *Comptes rendus - Biologies* **337**: 717–724.

Barbera G, Cullotta S, and Pizzurro GM. 2005. Agroforestry systems of Mt Etna, Italy: Biodiversity analysis at landscape, stand and specific level. *Monitoring and Indicators of Forest Biodiversity in Europe - from Ideas to Operationality* **51**: 481–492.

Barriga JC, Lassaletta L, and Moreno AG. 2010. Ground-living spider assemblages from Mediterranean habitats under different management conditions. *Journal of Arachnology* **38**: 258–269.

Boinot S, Fried G, Storkey J, *et al.* 2019a. Alley cropping agroforestry systems: Reservoirs for weeds or refugia for plant diversity? *Agriculture, Ecosystems and Environment* **284**: 106584.

Boinot S, Poulmarc'h J, Mézière D, *et al.* 2019b. Distribution of overwintering invertebrates in temperate agroforestry systems: Implications for biodiversity conservation and biological control of crop pests. *Agriculture, Ecosystems and Environment* **285**: 106630.

Buse J, Forest B, Park N, *et al.* 2014. Die Dungkäfer einer halboffenen Weidelandschaft mit einer Dauerbeweidung durch Rinder und Pferde. *Mainzer naturwissenschaftliches Archiv* **51**: 309–317.

Cardinael R, Hoeffner K, Chenu C, *et al.* 2019. Spatial variation of earthworm communities and soil organic carbon in temperate agroforestry. *Biology and Fertility of Soils* **55**: 171–183.

Cruz J, Sarmento P, Rydevik G, *et al.* 2016. Bats like vintage: managing exotic eucalypt plantations for bat conservation in a Mediterranean landscape. *Animal Conservation* **19**: 53–64.

da Silva PM, Aguiar CA, Niemelä J, *et al.* 2009. Cork-oak woodlands as key-habitats for biodiversity conservation in Mediterranean landscapes: A case study using rove and ground beetles (Coleoptera: Staphylinidae, Carabidae). *Biodiversity and Conservation* **18**: 605–619.

Debussche M, Debussche G, and Lepart J. 2001. Changes in the vegetation of Quercus pubescens woodland after cessation of coppicing and grazing. *Journal of Vegetation Science* **12**: 81–92.

Dorresteijn I, Hartel T, Hanspach J, *et al.* 2013. The conservation value of traditional rural landscapes: the case of woodpeckers in Transylvania, Romania. *PLoS ONE* **8**: e65236.

Fontana V, Radtke A, Walde J, *et al.* 2014. What plant traits tell us: Consequences of land-use change of a traditional agro-forest system on biodiversity and ecosystem service provision. *Agriculture, Ecosystems and Environment* **186**: 44–53.

Galle R, Urak I, Nikolett GS, and Hartel T. 2017. Sparse trees and shrubs confers a high biodiversity to pastures: Case study on spiders from Transylvania. *PloS one* **12**: e0183465.

García-Tejero S, Taboada Á, Tárrega R, and Salgado JM. 2013. Land use changes and ground dwelling beetle conservation in extensive grazing dehesa systems of north-west Spain. *Biological Conservation* **161**: 58–66.

Garrido-Jurado I, Fernández-bravo M, Campos C, and Quesada-moraga E. 2015. Diversity of entomopathogenic Hypocreales in soil and phylloplanes of five Mediterranean cropping systems. *Journal of Invertebrate Pathology* **130**: 97–106.

Gavazov K, Spiegelberger T, and Buttler A. 2014. Transplantation of subalpine wood-pasture turfs along a natural climatic gradient reveals lower resistance of unwooded pastures to climate change compared to wooded ones. *Oecologia* **174**: 1425–1435.

Giordani P, Incerti G, Rizzi G, *et al.* 2010. Land use intensity drives the local variation of lichen diversity in Mediterranean ecosystems sensitive to desertification. In: Nash TH (Ed.) Biology of Lichens – Symbiosis, Ecology, Environm. Monitoring, Systematics, Cyber Applications, 139–148. Stuttgart: J. Cramer in der Gebrüder Borntraeger Verlagsbuchhandlung.

Hartel T, Hanspach J, Abson DJ, and Máthé O. 2014. Bird communities in traditional wood-pastures with changing management in Eastern Europe. *Basic and Applied Ecology* **15**: 385–395.

Hill DA, Lambton S, Proctor I, *et al.* 1991. Winter bird communities in woodland in the forest of Dean, England, and some implications of livestock grazing. *Bird Study* **38**: 57–70.

Juutilainen K, Mönkkönen M, Kotiranta H, and Halme P. 2016. The role of novel forest ecosystems in the conservation of wood-inhabiting fungi in boreal broadleaved forests. *Ecology and Evolution* **6**: 6943–6954.

Listopad CMCS, Köbel M, Príncipe A, *et al.* 2018. The effect of grazing exclusion over time on structure, biodiversity, and regeneration of high nature value farmland ecosystems in Europe. *Science of the Total Environment* **610-611**: 926–936.

López-Carrasco C, López-Sánchez A, San Miguel A, and Roig S. 2015. The effect of tree cover on the biomass and diversity of the herbaceous layer in a Mediterranean dehesa. *Grass and Forage Science* **70**: 639–650.

López-Sánchez A, San A, Dirzo R, and Roig S. 2016a. Scattered trees and livestock grazing as keystones organisms for sustainable use and conservation of Mediterranean dehesas. *Journal for Nature Conservation* **33**: 58–67.

López-Sánchez A, San A, López-Carrasco C, *et al.* 2016b. The important role of scattered trees on the herbaceous diversity of a grazed Mediterranean dehesa. *Acta Oecologica* **76**: 31–38.

Mannu R, Pilia O, Leonarda M, and Marcello F. 2018. Variability of beetle assemblages in Mediterranean cork oak woodlands : does the higher taxa approach reliably characterize a specific response to grazing? *Biodiversity and Conservation* **27**: 3599–3619.

Mcadam JH, Sibbald AR, Teklehaimanot Z, and Eason WR. 2007. Developing silvopastoral systems and their effects on diversity of fauna. *Agroforestry Systems* **70**: 81–89.

Moreno G, Fernando GGb, and Diaz M. 2016. Exploring the causes of high biodiversity of Iberian dehesas: the importance of wood pastures and marginal habitats. *Agroforestry Systems* **90**: 87–105.

Morgan-Davies C, Waterhouse A, Pollock ML, and Holland JP. 2008. Integrating hill sheep production and newly established native woodland: achieving sustainability through multiple land use in Scotland. *International Journal of Agricultural Sustainability* **6**: 133–147.

Obrist MK, Rathey E, Bontadina F, *et al.* 2011. Response of bat species to sylvopastoral abandonment. *Forest Ecology and Management* **261**: 789–798.

Oldén A, Raatikainen KJ, Tervonen K, and Halme P. 2016. Grazing and soil pH are biodiversity drivers of vascular plants and bryophytes in boreal wood-pastures. *Agriculture Ecosystems & Environment* **222**: 171–184.

Paltto H, Nordberg A, Nordén B, and Snäll T. 2011. Development of secondary woodland in oak wood pastures reduces the richness of rare epiphytic lichens. *PloS one* **6**: e24675.

Pardon P, Reheul D, Mertens J, *et al.* 2019. Gradients in abundance and diversity of ground dwelling arthropods as a function of distance to tree rows in temperate

arable agroforestry systems. *Agriculture, Ecosystems and Environment* **270-271**: 114–128.

Peng RK, Incoll LD, Sutton SL, *et al.* 1993. Diversity of airborne arthropods in a silvoarable agroforestry system. *Journal of Applied Ecology* **30**: 551–562.

Peng RK and Sutton SL. 1996. The activity and diversity of ground arthropods in an agroforestry system. *Proceedings of the 49th New Zealand Plant Protection Conference* 309–313.

Pereira P, Godinho C, Gomes M, and Rabaça JE. 2014. The importance of the surroundings: are bird communities of riparian galleries influenced by agroforestry matrices in SW Iberian Peninsula ? *Annals of Forest Science* **71**: 33–41.

Razola I and Rey Benayas JM. 2009. Effects of woodland islets introduced in a Mediterranean agricultural landscape on local bird communities. *Web Ecology* **9**: 44–53.

Rösch V, Hoffmann M, Diehl U, and Entling MH. 2019. The value of newly created wood pastures for bird and grasshopper conservation. *Biological Conservation* .

Rossetti I, Bagella S, Cappai C, *et al.* 2015. Isolated cork oak trees affect soil properties and biodiversity in a Mediterranean wooded grassland. *Agriculture, Ecosystems and Environment* **202**: 203–216.

Slancarova J, Garcia-Pereira P, Fric ZF, *et al.* 2015. Butterflies in Portuguese 'montados': relationships between climate, land use and life-history traits. *Journal of Insect Conservation* **19**: 823–836.

Stoate C, Araújo M, and Borralho R. 2003. Conservation of European farmland birds: abundance and species diversity. *Ornis Hungarica* **12-13**: 33–40.

Taboada Á, Tárrega R, Calvo L, *et al.* 2010. Plant and carabid beetle species diversity in relation to forest type and structural heterogeneity. *European Journal of Forest Research* **129**: 31–45.

Tárrega R, Calvo L, Taboada Á, *et al.* 2009. Abandonment and management in Spanish dehesa systems: Effects on soil features and plant species richness and composition. *Forest Ecology and Management* **257**: 731–738.

Tölgyesi C, Bátori Z, Gallé R, *et al.* 2018. Shrub encroachment under the trees diversifies the herb layer in a Romanian silvopastoral system. *Rangeland Ecology and Management* **71**: 571–577.

Varah A. 2015. Can agroforestry reconcile conflicting demands for productivity , biodiversity conservation and delivery of ecosystem services ? Ph.D. thesis, University of Reading.

Wood TJ and Goulson D. 2017. The environmental risks of neonicotinoid pesticides: a review of the evidence post 2013. *Environmental Science and Pollution Research* **24**: 17285–17325.
