# Supplementary material for: European agroforestry has no unequivocal effect on biodiversity: a time-cumulative meta-analysis
Source: BMC Ecol Evol. 2021 Oct 23;21:193. doi: 10.1186/s12862-021-01911-9 (PMC8541809; doi:10.1186/s12862-021-01911-9)
Supplement: Supplementary file 5 — Additional file 5. Description of data tables and R-Code of the analysis. The file is in the R-Markdown format (.rmd) and can be opened via R-Studio or any other text editor. The Appendix 5 is also provided in the html format including the R-Output of the analysis. [file 12862_2021_1911_MOESM5_ESM.zip › 12862_2021_1911_MOESM5_ESM/Appendix5_Agroforestry_MetaAnalysis_Supplement.html]

Agroforestry Meta Analyses


# Agroforestry Meta Analyses

#### Anne Mupepele

#### 7/21/2017

This code was created in R version 4.0.2 (2020-06-22) with package versions up to date in July 2020.

```
library(car) #for recode()
library(RColorBrewer)
library(lattice)
library(grid) #for text in outer lattice margin
library(metafor) 
library(stringr) #Create unique labels for slab
library(nlme)
library(MASS) #stepAIC
library(dplyr) #function distinct (it masks the function recode from MASS!)
library(countrycode) # countrycode to link data to map shapefile
library(rworldmap) #background shape file for the map
library(plotrix)
library(ggplot2)
library(gridExtra) #grid.arrange for ggplot
library(MuMIn) #for pseudo R_squared (marginal R_squared)
library(tibble) # necessary for function to make effect size labels bold
library(gtable) # to label effect size plot
library(cowplot) # arranging plots
```

# Macros

## Define colours

Define colours for taxonomic groups used in colour-coded figures

```
arth.col <- "#173F5F"
bat.col <- "#20639B"
plant.col <- "#3CAEA3"
bird.col <- "#F6D55C"
flb.col <- "#f28875"
```

## Define functions

Combined standard deviation (GSD) (Square root of the ‘pooled variance’):

s=\(\sqrt{\dfrac{\displaystyle\sum\_{i=1}^{k}{(n\_i-1)s\_i^2+n\_i(\bar{x}\_i-\bar{x})^2}}{(\displaystyle\sum\_{i=1}^{k}{n\_i})-1}}\) (Duncan 1979)

```
GSD <- function(means,sds,ns){sqrt((sum((ns-1)*sds^2+ns*(means-mean(means))^2))/(sum(ns)-1))}
means <- c(4,5)
sds <- c(1,3)
ns <- c(10,11)
```

Highlighting y-axis label in ggplot to make summary effect size labels bold.

```
highlight <- function(x,y){
  exp <- vector(length = 0, mode = "expression")
  for (i in seq_along(x)) {
    if (i %in% y) exp[[i]] <- bquote(bold(.(x[i])))
    else exp[[i]] <- x[i]
  }
return(exp)
}
```

Extract the legend from ggplot to provide one legend for several plot

```
g_legend<-function(a.gplot){ 
  tmp <- ggplot_gtable(ggplot_build(a.gplot))
  leg <- which(sapply(tmp$grobs, function(x) x$name) == "guide-box")
  legend <- tmp$grobs[[leg]]
  return(legend)}
```

# Data

## Datasets

- ‘biodivdat’ are the raw data (86 rows and 29 columns) loaded from Meta\_Anal\_DataR\_Biodiv\_v4.csv
- ‘ef’ includes the cleaned raw data (cleaned by e.g. restructured factor levels) and the effect sizes (log response ratio). (86 rows and 36 columns) one row reflects a unique combinations of agroforestry system (silvoarable or silvopasture), control type (forest, cropland, pastures or abandoned and hence shrubby agroforestry systems) and taxonomic groups (*resolution as high as possible, e.g. one effect size for bees, one for butterflies*)
- ‘ef1’ includes the cleaned raw data and the effect sizes (log response ratio). (69 rows and 35 columns) one row reflects a unique combinations of agroforestry system (silvoarable or silvopasture), control type (forest, cropland, pastures or abandoned and hence shrubby agroforestry systems) and taxonomic groups (*low resolution: arthropods, birds, bats, plants, FLB (fungi, lichen plus bryophytes)*)
- ‘s’ are the data per study, i.e. one study = one row

Columns and their description

- Assessor - name of the author who has extracted the information from the study
- AuthorSnip - key for the primary studies where data were extracted from
- Title - title of the primary studies where data were extracted from
- Climate - climate zone according to (Walter and Breckle 1991)
- Climate1 - climate zone merging alpine studies with ‘temperate’ and atlantic studies (‘L’) with Mediterranean.
- Country - country in which the primary study site was located
- Agroforestry\_System - distinguishing slivopastoral systems from silvoarable systems
- Study\_design - study design in the primary study. One of: Case-Control, Before-After, Observational
- FinalLoE - the level of evidence according to the evidence assessment (Mupepele et al. 2016)
- Comparator - the ‘control type’ with which the agroforestry system was compared to calculate the effect size in the meta-analysis. The category ‘Agriculture’ was changed to ‘Cropland’ being a more suitable descriptor for agricultural crop fields.
- Dominant\_Tree\_Species - the dominant tree species in the agroforestry system
- Biodiversity\_Measure - the quantifiable measuer that was used to represent biodiversity. One of: species richness, family richness (number of families as mostly arthropods were not identified to species level), shannon diversity or log-series.
- Biodiversity\_Group - the taxonomic group investigated, reported as it was given in the primary study
- Biodiversity\_Group1 - restructuring the taxonomic levels into one of: ‘Arthropods,’ ‘Bats,’ ‘Birds,’ ‘Plants’ or ‘Fungi, Lichen and Bryophytes’
- Sampling\_Method - approach used to sample biodiversity, e.g. transects or pan-traps
- agrof\_mean agrof\_sd agrof\_n - Mean, standard deviation (sd) and sample size of biodiversity measured in agroforestry field(s)
- control\_mean control\_sd control\_n - Mean, standard deviation (sd) and sample size of biodiversity measured in the control site(s), which is specified in the columns ‘Comparator.’
- Sampling\_Year\_Start - the year in which the sampling took place or started
- Sampling\_Year\_End - the year in which the sampling ended if several years were sampled
- Comments - additional information on personal communication with authors or missing standard deviation and transformations.

```
biodivdat <- read.csv2("12862_2021_1911_MOESM4_ESM.csv",dec=".",sep=",",stringsAsFactors=T)
```

## Process data

Impute missing values and restructure category levels to create ‘ef.’

```
#Imputing missing standard deviation: Replace NA in the two column with the standard deviations with the mean across this column.
biodivdat$agrof_sd <- replace(biodivdat$agrof_sd,is.na(biodivdat$agrof_sd),mean(biodivdat$agrof_sd,na.rm=T))
biodivdat$control_sd<- replace(biodivdat$control_sd,is.na(biodivdat$control_sd),mean(biodivdat$control_sd,na.rm=T))

#Restructure category levels
biodivdat$Biodiversity_Group1  <- factor(car::recode(biodivdat$Biodiversity_Group,"c('Ants','Arthropods', 'Bees','Beetles','Bombus','Butterflies','Carabid','Collembola','Insects','Scarabaeoidea','Solitary Bees','Spiders','Staphylinid','Isopoda','Orthoptera and Mantidae','Diplopoda','Earthworms')='Arthropods';c('Birds','Passerine birds', 'Woodpeckers')='Birds';c('Plants','Vascular plants','Shrubs')='Plants';c('Fungi','Lichen','Bryophytes')='FLB'")) #Plants are now all vascular plants
biodivdat$Country <- factor(car::recode(biodivdat$Country,"c('UK-Northern Ireland', 'UK-Scotland', 'UK-Wales')='UK'"))
biodivdat$Dominant_Tree_Species <- gsub("([A-Za-z]+).*", "\\1", biodivdat$Dominant_Tree_Species)
biodivdat$Publication_Year <- as.numeric(str_extract_all(biodivdat$AuthorSnip, "[0-9]+"))
biodivdat$Climate1 <- factor(car::recode(biodivdat$Climate,"'L'='M';'Alpine'='N'"))
biodivdat$Comparator <- car::recode(biodivdat$Comparator,"'Forest_Plantation'='Forest';'Agriculture'='Cropland'")
#Impute the missing sampling year by using the publication year
biodivdat$Sampling_Year_Start[is.na(biodivdat$Sampling_Year_Start)] <-  str_extract(biodivdat$AuthorSnip, "\\-*\\d+\\.*\\d*")[is.na(biodivdat$Sampling_Year_Start)]
biodivdat$Sampling_Year_Start <- as.numeric(biodivdat$Sampling_Year_Start)
biodivdat$LoEsc <- as.numeric(as.character(car::recode(biodivdat$FinalLoE,"'LoE2b'=2.3;'LoE3a'=3;'LoE3b'=3.3;'LoE4a'=4;'LoE4b'=4.3")))
```

Create a data frame with log response ratio that will be used as effect size in the meta-analysis.

```
ef<-escalc(measure = "ROM", 
           m1i = agrof_mean, sd1i = agrof_sd, n1i = agrof_n, 
           m2i = control_mean, sd2i = control_sd, n2i = control_n, 
           data = biodivdat)
```

Combine effect sizes to have one effect size per combination of study, taxonomic group (according to Biodiversity\_Group1, i.e. 5 levels), agroforestry system and control type (comparator). Studies such as Varah 2015 have an effect size for butterflies and bees, which are both ‘arthropods’ and will be combined.

```
table(duplicated(ef[,names(ef)%in%c("AuthorSnip","Agroforestry_System", "Comparator","Biodiversity_Group1")])) #will remain with only 68 effect sizes
```

```
## 
## FALSE  TRUE 
##    69    14
```

```
ef$test <- paste(ef$AuthorSnip,ef$Agroforestry_System,ef$Comparator,ef$Biodiversity_Group1)
df <- data.frame(test=character(),
                agrof_mean_comb=numeric(),
                agrof_sd_comb=numeric(),
                agrof_n_comb=integer(),
                control_mean_comb=numeric(),
                control_sd_comb=numeric(),
                control_n_comb=integer(),
                stringsAsFactors=FALSE)#stringsASFactors= F to avoid that characters are converted to factors! This is the new default in R4.0.2

for(i in 1:as.numeric(table(duplicated(ef$test))[2])){                
tomerge <- ef[ef$test==ef$test[duplicated(ef$test)][i],]
df[i,1] <- tomerge$test[1]
df[i,2] <- mean(tomerge$agrof_mean)
df[i,3] <- GSD(tomerge$agrof_mean,tomerge$agrof_sd,tomerge$agrof_n)
df[i,4] <- sum(tomerge$agrof_n)
df[i,5] <- mean(tomerge$control_mean)
df[i,6] <- GSD(tomerge$control_mean,tomerge$control_sd,tomerge$control_n)
df[i,7] <- sum(tomerge$control_n)
}

dfchange <- df[duplicated(df$test)==F,]

efn <- ef[duplicated(ef$test)==F,] #throw the duplicates out of the full data set
#replace the values by new values

bn <- merge(efn,dfchange,by="test",all=T) #merge the old and the new data
bn$agrof_mean[is.na(bn$agrof_mean_comb)==F] <- bn$agrof_mean_comb[is.na(bn$agrof_mean_comb)==F]

ef1<-escalc(measure = "ROM", 
           m1i = agrof_mean, sd1i = agrof_sd, n1i = agrof_n, 
           m2i = control_mean, sd2i = control_sd, n2i = control_n, 
           data = bn[,2:34])

#data.frame(ef1$yi,efn[order(efn$AuthorSnip),]$yi,efn[order(efn$AuthorSnip),]$AuthorSnip,ef1$AuthorSnip) # to verify whether the new data frame matches the unchanged rows of the previous dataset 'ef'.
```

Create a data frame with one data entry (row) per study, instead of one per effect size, to analyse how many studies from one country are included etc.

```
s <- ef1 %>% distinct(AuthorSnip, .keep_all = TRUE)
table(s$Country[s$Climate1=="N"])
```

```
## 
##     Belgium     Finland      France     Germany       Italy    Portugal 
##           1           0           3           2           1           0 
##     Romania       Spain      Sweden Switzerland      Turkey          UK 
##           4           0           0           2           0           6
```

```
table(s$Climate1)
```

```
## 
##  B  M  N 
##  4 27 19
```

# Descriptive analysis

## Figure Descriptive

Descriptive figure to display number of effect sizes per category.

```
d <- data.frame(table(paste(ef1[,names(ef1)=="Agroforestry_System"],ef1[,names(ef1)=="Comparator"],ef1[,names(ef1)=="Biodiversity_Group1"],sep=".")))
ef1_ggplot <- data.frame(d$Freq,t(data.frame(strsplit(as.character(d$Var1),split=".",fixed=T))),row.names = NULL)
names(ef1_ggplot) <- c("Freq","Agroforestry_System","Comparator","Biodiversity_Group1")
ef1_ggplot$Agroforestry_System <- factor(ef1_ggplot$Agroforestry_System,levels=c("Silvopasture","Silvoarable"))
ef1_ggplot$Comparator<- factor(car::recode(ef1_ggplot$Comparator,"c('Cropland','Pasture')='Cropland/Pasture'"),levels=c("Cropland/Pasture","Forest","Abandoned"))
ef1_ggplot$Biodiversity_Group1<- factor(ef1_ggplot$Biodiversity_Group1,levels=c("Plants","FLB", "Bats","Birds","Arthropods"))

#replace FLB with "Fungi, Lichens,\n& Bryophytes"
ef1_ggplot$Biodiversity_Group1 <- as.character(ef1_ggplot$Biodiversity_Group1)
ef1_ggplot$Biodiversity_Group1[ef1_ggplot$Biodiversity_Group1 == "FLB"] <- "Fungi, Lichens, & Bryophytes"
ef1_ggplot$Biodiversity_Group1 <- as.factor(ef1_ggplot$Biodiversity_Group1)

#bring in order for plotting
positions <- c("Plants", "Fungi, Lichens, & Bryophytes", "Bats", "Birds", "Arthropods")

nreff <- ggplot(ef1_ggplot, aes(x=Biodiversity_Group1, y=Freq, fill=Biodiversity_Group1)) +
  geom_bar(stat="identity", position=position_dodge()) + 
  scale_fill_manual("", values = c("Arthropods" = arth.col, "Birds" = bird.col, "Bats" = bat.col, "Fungi, Lichens, & Bryophytes" = flb.col, "Plants" = plant.col))+
  scale_x_discrete(limits = positions, labels = c("Plants", "Fungi, Lichens,\n& Bryophytes ", "Bats", "Birds", "Arthropods"))+
  labs(y="Number of effects sizes", x="Taxonomic group")+
  scale_y_continuous(limits = c(0, 10), breaks = seq(0, 10, by = 2))+
  theme_minimal() +
  theme(axis.title.y  = element_text(face="bold", vjust = 2, size = 13),
        axis.title.x  = element_text(face="bold", vjust = -1, size = 13),
        strip.text = element_text(face="bold"),
        legend.title = element_text(face="bold"),
        panel.grid.minor = element_blank(),
        panel.grid.major.y = element_blank(),
        legend.position = "none") +
  facet_grid(Comparator~Agroforestry_System)+
  coord_flip()

# add titles "Comparator" and "Agroforestry System"
dev.control(displaylist="enable")
nreff + theme(plot.margin = unit(c(1.5,1.5,0.2,0.2), "cm"))
grid::grid.text(unit(0.95,"npc"),unit(0.483,'npc'),label = 'Control type', rot = 270, gp=gpar(fontsize=13, fontface="bold"))# right 
grid::grid.text(unit(0.515,"npc"),unit(.94,'npc'),label = 'Agroforestry System', rot = 0, gp=gpar(fontsize=13, fontface="bold"))   # top
```

```
effectsizes_plot <- recordPlot()
```

## Map

```
#per effect sizes
country_coded <- countrycode(names(sort(table(ef1$Country))), 'country.name', 'iso3c')

map_data <- data.frame(country = country_coded,
  effect_sizes = as.numeric(sort(table(ef1$Country))))
map_data_coun <- joinCountryData2Map(map_data, joinCode = "ISO3",
  nameJoinColumn = "country")# join map_data data.frame to the country map data
```

```
## 12 codes from your data successfully matched countries in the map
## 0 codes from your data failed to match with a country code in the map
## 231 codes from the map weren't represented in your data
```

```
dev.control(displaylist="enable")
map <- mapCountryData(map_data_coun, mapRegion = "Europe", nameColumnToPlot="effect_sizes", catMethod = "categorical", missingCountryCol = gray(.8), lwd=1.2, colourPalette = brewer.pal(9,"Blues"), mapTitle = "", addLegend = FALSE) #plot the map
do.call(addMapLegendBoxes, c(map, title = "Number of effect sizes per country", horiz = TRUE, x='bottom',bty="o",col="black",cex=0.7))
```

```
map <- recordPlot()
```

# Meta-Analysis

## Model selection

Model with a random effect on ‘study’ (variable name: AuthorSnip) and no fixed effects. R-package ‘metafor’ and r-package ‘nlme’ lead to the same conclusions (compare model ‘estimate’ with ‘intercept’ and ‘sigma2 sqrt’ with random effects ‘StdDev’).

```
rma.mv(yi,vi,random=~1|AuthorSnip,data=ef1,method="ML")
```

```
## 
## Multivariate Meta-Analysis Model (k = 69; method: ML)
## 
## Variance Components:
## 
##             estim    sqrt  nlvls  fixed      factor 
## sigma^2    0.1879  0.4334     50     no  AuthorSnip 
## 
## Test for Heterogeneity:
## Q(df = 68) = 5809.5134, p-val < .0001
## 
## Model Results:
## 
## estimate      se    zval    pval    ci.lb   ci.ub 
##   0.0986  0.0659  1.4953  0.1348  -0.0306  0.2279    
## 
## ---
## Signif. codes:  0 '***' 0.001 '**' 0.01 '*' 0.05 '.' 0.1 ' ' 1
```

```
lme(yi~1,random=~1|AuthorSnip,weights=varFixed(~vi),control=list(sigma=1),method="ML",data=ef1)
```

```
## Linear mixed-effects model fit by maximum likelihood
##   Data: ef1 
##   Log-likelihood: -293.856
##   Fixed: yi ~ 1 
## (Intercept) 
##   0.0986048 
## 
## Random effects:
##  Formula: ~1 | AuthorSnip
##         (Intercept) Residual
## StdDev:   0.4334361        1
## 
## Variance function:
##  Structure: fixed weights
##  Formula: ~vi 
## Number of Observations: 69
## Number of Groups: 50
```

Fixed effects: Model selection with Likelihood-Ratio test (based on ANOVA (F-Statistic))

```
mfull_lme <- lme(yi~Agroforestry_System+Biodiversity_Group1+Climate1+Comparator+Sampling_Year_Start,
             random=~1|AuthorSnip,
             weights=varFixed(~vi),
             control=list(sigma=1),
             method="ML",
             data=ef1)
anova(mfull_lme)
```

```
##                     numDF denDF  F-value p-value
## (Intercept)             1    47  2.03824  0.1600
## Agroforestry_System     1    10 53.32176  <.0001
## Biodiversity_Group1     4    10 22.09757  0.0001
## Climate1                2    47  0.98256  0.3819
## Comparator              3    10  6.38381  0.0109
## Sampling_Year_Start     1    10  0.14974  0.7069
```

```
anova(mfull_lme,m1_lme <- update(mfull_lme,~.-Climate1))
```

```
##                                            Model df      AIC      BIC    logLik
## mfull_lme                                      1 13 416.6189 445.6623 -195.3095
## m1_lme <- update(mfull_lme, ~. - Climate1)     2 11 414.3977 438.9729 -196.1988
##                                              Test  L.Ratio p-value
## mfull_lme                                                         
## m1_lme <- update(mfull_lme, ~. - Climate1) 1 vs 2 1.778758  0.4109
```

```
anova(m1_lme)
```

```
##                     numDF denDF  F-value p-value
## (Intercept)             1    49  2.02151  0.1614
## Agroforestry_System     1    10 55.47817  <.0001
## Biodiversity_Group1     4    10 22.81079  0.0001
## Comparator              3    10  6.68287  0.0094
## Sampling_Year_Start     1    10  0.29148  0.6011
```

```
anova(m1_lme,m2_lme <- update(m1_lme,~.-Sampling_Year_Start))
```

```
##                                                    Model df      AIC      BIC
## m1_lme                                                 1 11 414.3977 438.9729
## m2_lme <- update(m1_lme, ~. - Sampling_Year_Start)     2 10 412.7339 435.0750
##                                                       logLik   Test  L.Ratio
## m1_lme                                             -196.1988                
## m2_lme <- update(m1_lme, ~. - Sampling_Year_Start) -196.3669 1 vs 2 0.336206
##                                                    p-value
## m1_lme                                                    
## m2_lme <- update(m1_lme, ~. - Sampling_Year_Start)   0.562
```

```
anova(m2_lme)
```

```
##                     numDF denDF  F-value p-value
## (Intercept)             1    49  2.02319  0.1612
## Agroforestry_System     1    11 56.52516  <.0001
## Biodiversity_Group1     4    11 23.17437  <.0001
## Comparator              3    11  6.78374  0.0074
```

```
anova(m2_lme,m3_lme <- update(m2_lme,~.-Comparator))
```

```
##                                           Model df      AIC     BIC    logLik
## m2_lme                                        1 10 412.7339 435.075 -196.3669
## m3_lme <- update(m2_lme, ~. - Comparator)     2  7 430.0482 445.687 -208.0241
##                                             Test  L.Ratio p-value
## m2_lme                                                           
## m3_lme <- update(m2_lme, ~. - Comparator) 1 vs 2 23.31432  <.0001
```

## R2 - Heterogeneity explained by the fixed effects.

Heterogeneity (between-study variability) explained by the model.

Marginal R values as in Martin et al. (2015), given by Nakagawa and Schielzeth (2013).

```
r.squaredGLMM(m2_lme)[[1]]
```

```
## [1] 0.04239325
```

# Forest plots - subgroup analysis

## Silvopasture subgroup forest plot

```
rma_intercept <- rma.mv(yi, vi, data = ef1, method = "ML",random = list(~ 1 | AuthorSnip))

#Silvopasture
dat1 <- droplevels(ef1[ef1$Agroforestry_System=="Silvopasture",][,names(ef1)%in%c("AuthorSnip","Comparator","Biodiversity_Group1","yi","vi")])
dat1$ci.lb <- summary(dat1)$ci.lb
dat1$ci.ub <- summary(dat1)$ci.ub
dat1$Type <- as.factor(rep("EffectSize",nrow(dat1)))

rma_silvopasture <- rma.mv(yi, vi, data = dat1, method = "ML",random = list(~ 1 | AuthorSnip))
model <- vector("list",length(levels(dat1$Comparator))) #empty list of length() elements
for (i in 1:length(levels(dat1$Comparator))){ 
  model[[i]] <- rma.mv(yi,vi,subset=(Comparator==levels(Comparator)[i]),data=dat1, method="ML",random=list(~1|AuthorSnip))
}
names(model) <- levels(dat1$Comparator)

dat2 <- data.frame(AuthorSnip=paste(levels(dat1$Comparator),rep(" summary effect size",3),sep=""),Comparator=levels(dat1$Comparator),Biodiversity_Group1=rep("",3),yi=sapply(1:3,function(i)as.numeric(model[[i]]$beta)),vi=as.numeric(rep(NA,3)),ci.lb=sapply(1:3,function(i)as.numeric(model[[i]]$ci.lb)),ci.ub=sapply(1:3,function(i)as.numeric(model[[i]]$ci.ub)),Type=rep("Summary",3))

dat3 <- data.frame(AuthorSnip=rep("Silvopasture summary effect size",1),Comparator=rep("",1),Biodiversity_Group1=rep("",1),yi=as.numeric(rma_silvopasture$beta), vi=as.numeric(rep(NA,1)),ci.lb=as.numeric(rma_silvopasture$ci.lb),ci.ub=as.numeric(rma_silvopasture$ci.ub),Type=rep("GrandSummary",1))

dat1 <- lapply(dat1, function(x) if(is.factor(x)) as.character(x) else x)
dat2 <- lapply(dat2, function(x) if(is.factor(x)) as.character(x) else x)
dat3 <- lapply(dat3, function(x) if(is.factor(x)) as.character(x) else x)

dat <- rbind(data.frame(dat1),data.frame(dat2),data.frame(dat3))
dat$Comparator <- factor(dat$Comparator,levels=c("Pasture","Forest","Abandoned"))

dat <- dat[order(factor(dat$Type,level=c("EffectSize","Summary","GrandSummary")),factor(dat$Comparator,level=c("Pasture","Forest","Abandoned")),factor(dat$Biodiversity_Group1)),]

# add numbered label for order of studies
dat$label <- paste(nrow(dat):1+10) 

# add multiple as biodiversity group category for summary effect size
dat$Biodiversity_Group1 <- factor(dat$Biodiversity_Group1,levels = c("Arthropods","Bats","Birds", "FLB", "Plants", ""))
levels(dat$Biodiversity_Group1)[levels(dat$Biodiversity_Group1) == ""] <- "Multiple"

# remove NA for grey box of overall effect
dat$Comparator <- factor(dat$Comparator, levels = levels(addNA(dat$Comparator)), labels = c(levels(dat$Comparator), " "), exclude = NULL) 

# define colours
cols6 = c(arth.col, bat.col, bird.col, flb.col, plant.col, "grey")

#data for diamonds as summary effect size
sub <- dat[dat$Type%in%c("Summary","GrandSummary"),]
diamonds <- data.frame(
  x=c(sapply(1:4,function(i)c(sub[i,]$ci.lb,sub[i,]$yi,sub[i,]$ci.ub,sub[i,]$yi))),
  y=rep(c(1,0.6,1,1.4),4),
  Comparator=factor(rep(levels(sub$Comparator),each=4)),Biodiversity_Group1=factor(rep("Multiple",16),levels = c("Arthropods","Bats","Birds", "FLB", "Plants", "Multiple")))

# actual plot (from Dominic modified from Anne)
silvopasture_forest <- ggplot(dat, aes(y=label, x=yi, xmin=ci.lb, xmax=ci.ub, color=Biodiversity_Group1, fill=Biodiversity_Group1)) +
  geom_vline(xintercept=0, color='black', linetype='dashed')+   #Add a vertical dashed line indicating an effect size of zero, for reference
  geom_errorbarh(size=0.8,position=position_dodge(width = 0.5), alpha=1,height=0) +
geom_point(data=subset(dat, Type=='EffectSize'),size=0.8,stat= "identity", color = 'black')+ #Add data points and color them black
  geom_point(data=subset(dat, Type!='EffectSize'), color='white')+ #Add 'special' points for the summary estimates
  geom_errorbarh(data=subset(dat, Type!='EffectSize'),size=0.9,position=position_dodge(width = 0.5), alpha=1,height=0,color='white')+
  geom_polygon(data = diamonds,aes(x = x, y = y,fill=Biodiversity_Group1),inherit.aes=F,color="grey")+ #add summary diamond
  scale_x_continuous(limits=c(-2.5,4), name='Log Response Ratio')+ #Specify the limits of the x-axis and relabel it to something more meaningful
  scale_y_discrete(breaks = dat$label, labels = highlight(as.character(dat$AuthorSnip),c(53,54,55,56)))+ # numbers refer to row numbers of dat
  ylab('Reference')+ #Give y-axis a meaningful label
 facet_grid(Comparator~., scales= 'free', space='free')+ #Create sub-plots (i.e., facets) based on levels of Comparator, I could change label here ,labeller=hospital_labeller. And allow them to have their own unique axes (so authors don't redundantly repeat)
  ggtitle("Silvopasture")+
  scale_fill_manual(values=cols6, guide = F)+
  scale_color_manual(values=cols6, name = "Taxonomic group", labels=c("Arthropods","Bats","Birds", "Fungi, Lichens,\n& Bryophytes", "Plants", "Multiple"))+ # legend title
  theme_bw()+
  theme(panel.grid.major=element_blank(),
        panel.grid.minor=element_blank(),
        panel.border=element_blank(),
        axis.line=element_line(),
        axis.text.y = element_text(size=7),
        axis.title = element_text(size=8,face ="bold"),
        strip.background = element_rect(colour="white", fill="white"), #strip refers to the facet labels, but can only change them all at ones.
        legend.position='right')

#remove the grey background on the last facet label that characterizes only the silvoarable summary effect size
g1 <- ggplot_gtable(ggplot_build(silvopasture_forest+theme(legend.position="none"))) #create gtable from modified (without legend) silvopasture_forest ggplot
stripr <- which(grepl('strip-r', g1$layout$name))
fills <- c("grey","grey","grey","white")
k <- 1
for (i in stripr) {
j <- which(grepl('rect', g1$grobs[[i]]$grobs[[1]]$childrenOrder))
g1$grobs[[i]]$grobs[[1]]$children[[j]]$gp$fill <- fills[k]
k <- k+1
}
```

## Silvoarable subgroup forest plot

```
rma_intercept <- rma.mv(yi, vi, data = ef1, method = "ML",random = list(~ 1 | AuthorSnip))

#Silvoarable
dat1 <- droplevels(ef1[ef1$Agroforestry_System=="Silvoarable",][,names(ef1)%in%c("AuthorSnip","Comparator","Biodiversity_Group1","yi","vi")])
dat1$ci.lb <- summary(dat1)$ci.lb
dat1$ci.ub <- summary(dat1)$ci.ub
dat1$Type <- as.factor(rep("EffectSize",nrow(dat1)))

rma_silvoarable <- rma.mv(yi, vi, data = dat1, method = "ML",random = list(~ 1 | AuthorSnip))
model <- vector("list",length(levels(dat1$Comparator))) #empty list of length() elements
for (i in 1:length(levels(dat1$Comparator))){ 
  model[[i]] <- rma.mv(yi,vi,subset=(Comparator==levels(Comparator)[i]),data=dat1, method="ML",random=list(~1|AuthorSnip))
}
names(model) <- levels(dat1$Comparator)

dat2 <- data.frame(AuthorSnip=paste(levels(dat1$Comparator),rep(" summary effect size",3),sep=""),Comparator=levels(dat1$Comparator),Biodiversity_Group1=rep("",3),yi=sapply(1:3,function(i)as.numeric(model[[i]]$beta)),vi=as.numeric(rep(NA,3)),ci.lb=sapply(1:3,function(i)as.numeric(model[[i]]$ci.lb)),ci.ub=sapply(1:3,function(i)as.numeric(model[[i]]$ci.ub)),Type=rep("Summary",3))

dat3 <- data.frame(AuthorSnip=rep("Silvoarable summary effect size",1),Comparator=rep("",1),Biodiversity_Group1=rep("",1),yi=as.numeric(rma_silvoarable$beta),vi=as.numeric(rep(NA,1)),ci.lb=as.numeric(rma_silvoarable$ci.lb),ci.ub=as.numeric(rma_silvoarable$ci.ub),Type=rep("GrandSummary",1))

dat1 <- lapply(dat1, function(x) if(is.factor(x)) as.character(x) else x)
dat2 <- lapply(dat2, function(x) if(is.factor(x)) as.character(x) else x)
dat3 <- lapply(dat3, function(x) if(is.factor(x)) as.character(x) else x)

dat <- rbind(data.frame(dat1),data.frame(dat2),data.frame(dat3))
dat$Comparator <- factor(dat$Comparator,levels=c("Cropland","Forest","Abandoned"))

dat <- dat[order(factor(dat$Type,level=c("EffectSize","Summary","GrandSummary")),factor(dat$Comparator,level=c("Cropland","Forest","Abandoned")),factor(dat$Biodiversity_Group1)),]

# add label to order studies
dat$label <- paste(nrow(dat):1+10) 

# add multiple as biodiversity group category for summary effect size
dat$Biodiversity_Group1 <- factor(dat$Biodiversity_Group1,levels = c("Arthropods","Birds", "FLB", "Plants", ""))
levels(dat$Biodiversity_Group1)[levels(dat$Biodiversity_Group1) == ""] <- "Multiple"

# remove NA for grey box of overall effect
dat$Comparator <- factor(dat$Comparator, levels = levels(addNA(dat$Comparator)), labels = c(levels(dat$Comparator), " "), exclude = NULL) 

# define colours
cols = c(arth.col, bird.col, flb.col, plant.col, "grey") # no bat studies in silvoarable - one colour less

#data for diamonds as summary effect size
sub <- dat[dat$Type%in%c("Summary","GrandSummary"),]
diamonds <- data.frame(
  x=c(sapply(1:4,function(i)c(sub[i,]$ci.lb,sub[i,]$yi,sub[i,]$ci.ub,sub[i,]$yi))),
  y=rep(c(1,0.8,1,1.2),4),
 Comparator=factor(rep(levels(sub$Comparator),each=4)),Biodiversity_Group1=factor(rep("Multiple",16),levels = c("Arthropods","Birds", "FLB", "Plants", "Multiple")))

# actual plot (from Dominic modified from Anne)
silvoarable_forest <- ggplot(dat, aes(y=label, x=yi, xmin=ci.lb, xmax=ci.ub, color=Biodiversity_Group1, fill=Biodiversity_Group1)) +
  geom_vline(xintercept=0, color='black', linetype='dashed')+   #Add a vertical dashed line indicating an effect size of zero, for reference
  geom_errorbarh(size=0.8,position=position_dodge(width = 0.5), alpha=1,height=0) +
geom_point(data=subset(dat, Type=='EffectSize'),size=0.8,stat= "identity", color = 'black')+ #Add data points and color them black
  geom_point(data=subset(dat, Type!='EffectSize'), color='white')+ #Add 'special' points for the summary estimates
  geom_errorbarh(data=subset(dat, Type!='EffectSize'),size=0.9,position=position_dodge(width = 0.5), alpha=1,height=0,color='white')+
  geom_polygon(data = diamonds,aes(x = x, y = y,fill=Biodiversity_Group1),inherit.aes=F)+ #add summary diamond
  scale_x_continuous(limits=c(-1.5,4), name='Log Response Ratio')+ #Specify the limits of the x-axis and relabel it to something more meaningful
  scale_y_discrete(breaks = dat$label, labels = highlight(as.character(dat$AuthorSnip),c(18,19,20,21)))+ # numbers refer to row numbers of dat
  ylab('Reference')+ #Give y-axis a meaningful label
  facet_grid(Comparator~., scales= 'free', space='free')+ #Create sub-plots (i.e., facets) based on levels of Comparator, I could change label here ,labeller=hospital_labeller. And allow them to have their own unique axes (so authors don't redundantly repeat)
  ggtitle("Silvoarable")+
  scale_fill_manual(values=cols, guide = F)+
  scale_color_manual(values=cols, name = "Taxonomic group", labels=c("Arthropods","Birds", "Fungi, Lichens,\n& Bryophytes", "Plants", ""))+ # legend title, empty field in label to remove 'multiple' from the legend
  theme_bw()+
  theme(panel.grid.major=element_blank(),
        panel.grid.minor=element_blank(),
        panel.border=element_blank(),
        axis.line=element_line(),
        axis.text.y = element_text(size=7),
        axis.title = element_text(size=8,face ="bold"),
        strip.background = element_rect(colour="white",fill=NA), #strip refers to the facet labels, but can only change them all at ones.
        legend.position='right')

#remove the grey background on the last facet label that characterizes only the silvoarable summary effect size
g2 <- ggplot_gtable(ggplot_build(silvoarable_forest+theme(legend.position="none",axis.title.y=element_blank()))) #create gtable from modified (remove legend and axis) silvoarable_forest ggplot
stripr <- which(grepl('strip-r', g2$layout$name))
fills <- c("grey","grey","grey","white")
k <- 1
for (i in stripr) {
j <- which(grepl('rect', g2$grobs[[i]]$grobs[[1]]$childrenOrder))
g2$grobs[[i]]$grobs[[1]]$children[[j]]$gp$fill <- fills[k]
k <- k+1
}
```

Translating the summary effect size of the Cropland subgroup analysis in the impact on biodiversity to estimate the magnitude of the effect.

```
exp(model$Cropland$beta)
```

```
##             [,1]
## intrcpt 1.481903
```

```
with(ef1[ef1$Comparator=="Cropland",],mean(control_mean))
```

```
## [1] 5.496667
```

On one species in the agricultural control site, there will be 1.6 species in the agroforestry site. With an average of five species in the control site this leads to 8 species in the agroforestry site.

### Combined forest plot

Combine the two subplots silvopasture and silvoarable and use a shared legend.

```
layoutmatrix <- rbind(c(1,1,2,2),
             c(1,1,2,2),
             c(1,1,2,2),
             c(1,1,2,2),
             c(1,1,2,2),
             c(1,1,2,2),
             c(1,1,2,2),
             c(3,3,3,3))

cols6 = c(arth.col, bat.col, bird.col, flb.col, plant.col, "grey")

#g1 is the silvopasture_forest ggplot with modified facet background colors
#g2 is the silvoarable_forest ggplot with modified facet background colors

sharedlegend <- g_legend(silvopasture_forest+theme(legend.position = 'bottom')+ theme(legend.title=element_blank())+scale_color_manual(values=cols6, name = "Taxonomic group", labels=c("Arthropods", "Bats", "Birds", "Fungi,  Lichens,\n& Bryophytes","Plants"),breaks=c("Arthropods","Bats","Birds", "FLB", "Plants")))

#cairo_pdf(file="Figures/Figure_Forest_Plot.pdf",width =11,height =7)
grid.arrange(grobs=list(g1,g2,sharedlegend),layout_matrix = layoutmatrix)
```

```
#dev.off()
```

# Arthropods

More detailed subgroup analysis for arthropods with effect sizes based on ‘ef’ (i.e. higher resolution without lumping all arthropods per study, but separate effect sizes e.g. for beetles and bees).

```
arthropods <- droplevels(ef[ef$Biodiversity_Group1=="Arthropods",])
arthropods$arthropod_group_detailed <- factor(car::recode(arthropods$Biodiversity_Group,"c('Ants')='02_Ants';c('Bees','Bombus','Solitary Bees')='02_Bees';c('Beetles')='01_Beetles';c('Carabid')='01_Carabid';c('Staphylinid')='01_Staphylinid';c('Scarabaeoidea')='01_Scarabaeoid';c('Butterflies')='03_Butterflies';c('Spiders')='04_Spiders';c('Orthoptera and Mantidae')='05_Orthoptera and Mantidae';c('Isopoda')='06_Isopoda';c('Collembola')='07_Collembola';c('Diplopoda')='08_Diplopoda';c('Insects','Arthropods')='09_Arthropods';c('Earthworms')='10_Annelids'")) 

table(duplicated(arthropods[,names(arthropods)%in%c("AuthorSnip","Agroforestry_System", "Comparator","arthropod_group_detailed")]))
```

```
## 
## FALSE 
##    37
```

```
#data.frame(arthropods$yi,efn[order(efn$AuthorSnip),]$yi,efn[order(efn$AuthorSnip),]$AuthorSnip,arthropods$AuthorSnip) #to verify whether the new data frame matches the unchanged rows of the previous dataset 'efn'.
```

## Forest plot for arthropods

```
rma.mv(yi, vi, data = droplevels(ef1[ef1$Biodiversity_Group1=="Arthropods",]), method = "ML",random = list(~ 1 | AuthorSnip))
```

```
## 
## Multivariate Meta-Analysis Model (k = 27; method: ML)
## 
## Variance Components:
## 
##             estim    sqrt  nlvls  fixed      factor 
## sigma^2    0.1674  0.4092     21     no  AuthorSnip 
## 
## Test for Heterogeneity:
## Q(df = 26) = 566.3009, p-val < .0001
## 
## Model Results:
## 
## estimate      se    zval    pval   ci.lb   ci.ub 
##   0.2248  0.0947  2.3748  0.0176  0.0393  0.4103  * 
## 
## ---
## Signif. codes:  0 '***' 0.001 '**' 0.01 '*' 0.05 '.' 0.1 ' ' 1
```

```
rma_arthropods <- rma.mv(yi, vi, data = arthropods, method = "ML",random = list(~ 1 | AuthorSnip))
rma_arthropods_sp <- rma.mv(yi, vi, subset=(Agroforestry_System=="Silvopasture"), data = arthropods, method = "ML",random = list(~ 1 | AuthorSnip)) # silvopasture
rma_arthropods_sa <- rma.mv(yi, vi, subset=(Agroforestry_System=="Silvoarable"), data = arthropods, method = "ML",random = list(~ 1 | AuthorSnip)) # silvoarable

#Arthropods
dat1 <- droplevels(arthropods[,names(arthropods)%in%c("AuthorSnip","Agroforestry_System","Comparator","arthropod_group_detailed","yi","vi")])
dat1$ci.lb <- summary(dat1)$ci.lb
dat1$ci.ub <- summary(dat1)$ci.ub
dat1$Type <- as.factor(rep("EffectSize",nrow(dat1)))

# extract relevant data from models (for both types and then for silvopasture and silvoarable separately)
 dat3 <- data.frame(AuthorSnip=rep("Summary effect size",1),Agroforestry_System=rep("",1),Comparator=rep("",1),arthropod_group_detailed=rep("",1), yi=as.numeric(rma_arthropods$beta),vi=as.numeric(rep(NA,1)),ci.lb=as.numeric(rma_arthropods$ci.lb),ci.ub=as.numeric(rma_arthropods$ci.ub),Type=rep("GrandSummary",1))
 dat3.2 <- data.frame(AuthorSnip=rep("Silvopasture summary effect size",1),Agroforestry_System=rep("Silvopasture",1), Comparator=rep("",1),arthropod_group_detailed=rep("",1), yi=as.numeric(rma_arthropods_sp$beta),vi=as.numeric(rep(NA,1)),ci.lb=as.numeric(rma_arthropods_sp$ci.lb),ci.ub=as.numeric(rma_arthropods_sp$ci.ub),Type=rep("SilvopastureSummary",1))
  dat3.3 <- data.frame(AuthorSnip=rep("Silvoarable summary effect size",1),Agroforestry_System=rep("Silvoarable",1),Comparator=rep("",1), arthropod_group_detailed=rep("",1), yi=as.numeric(rma_arthropods_sa$beta),vi=as.numeric(rep(NA,1)),ci.lb=as.numeric(rma_arthropods_sa$ci.lb),ci.ub=as.numeric(rma_arthropods_sa$ci.ub),Type=rep("SilvoarableSummary",1))
  
# change factor/character
 dat1 <- lapply(dat1, function(x) if(is.factor(x)) as.character(x) else x)
 dat3 <- lapply(dat3, function(x) if(is.factor(x)) as.character(x) else x)

 dat3.2 <- lapply(dat3.2, function(x) if(is.factor(x)) as.character(x) else x)
 dat3.3 <- lapply(dat3.3, function(x) if(is.factor(x)) as.character(x) else x)

dat <- rbind(data.frame(dat1),data.frame(dat3),data.frame(dat3.2),data.frame(dat3.3))
dat$Comparator <- factor(dat$Comparator,levels=c("Cropland","Pasture","Forest","Abandoned"))
dat$Agroforestry_System <- factor(dat$Agroforestry_System,levels=c("Silvopasture",  "Silvoarable",""))

dat <- dat[order(factor(dat$Type,level=c("EffectSize","SilvoarableSummary","SilvopastureSummary","GrandSummary")),dat$Comparator,dat$arthropod_group_detailed),]

# add label to order studies
dat$label <- paste(nrow(dat):1+10) 

# define colours
cols <- c(gray.colors(5)[c(1,1,4)],'green',"black")

# summary effect size in diamond shape
sub <- dat[dat$Type%in%c("SilvoarableSummary","SilvopastureSummary","GrandSummary"),]
diamonds <- data.frame(
  x=c(sapply(1:3,function(i)c(sub[i,]$ci.lb,sub[i,]$yi,sub[i,]$ci.ub,sub[i,]$yi))),
  y=rep(c(1,0.8,1,1.2),3),
  SummaryColumn=factor(rep("",12)),
  Agroforestry_System=factor(rep(levels(factor(sub$Agroforestry_System)),each=4)),levels=c("Silvopasture",  "Silvoarable","")) #this column is required for the right arrangement of the diamonds on the y-scale (according to facets)
arthropods_mod <- dat

# actual plot
arthropods_forest <- ggplot(dat, aes(y=label, x=yi, xmin=ci.lb, xmax=ci.ub))+#, color = Comparator, fill=Comparator))+ #I could color according to Comparator, but then overloaded
  geom_vline(xintercept=0, color='black', linetype='dashed')+   #Add a vertical dashed line indicating an effect size of zero, for reference
   geom_errorbarh(data=subset(dat, Type=='EffectSize'),size=0.8,position=position_dodge(width = 0.5), alpha=1,height=0,color=arth.col) + 
  geom_point(data=subset(dat, Type=='EffectSize'),stat= "identity", color = 'black')+ #Add effect sizes as black points 
 # geom_point(data=subset(dat, Type%in%c('GrandSummary','SilvopastureSummary','SilvoarableSummary')), colour='pink',size=0.2,shape=3)+ #pink crosses for summaries, overwritten by polygon if polygon is non-transparent (alpha=1)
    geom_polygon(data = diamonds,aes(x = x, y = y,fill=SummaryColumn),inherit.aes=F,color='grey',fill='grey')+ #add summary diamond
  geom_text(aes(label=substr(arthropod_group_detailed,4,29),x=-2.4),size=3,color='black',hjust=0)+#hjust=0 for left-alignment
  geom_text(aes(label=substr(Comparator,1,1),x=2),size=3,color='black',hjust=0)+#hjust=0 for left-alignment
  scale_x_continuous(limits=c(-2.5,2), name='Log Response Ratio')+ #Specify the limits of the x-axis and relabel it to something more meaningful
   scale_y_discrete(breaks = dat$label, labels = highlight(as.character(dat$AuthorSnip),c(42, 43,44)))+ # numbers refer to row numbers of dat
  ylab('Reference')+ #Give y-axis a meaningful label
 facet_grid(Agroforestry_System~., scales= 'free', space='free')+ 
     scale_fill_manual(values=cols, guide = F)+
  #scale_color_manual(values=cols, name="",labels=c(levels(dat$Comparator),""))+ # legend title
  theme_bw()+
  theme(panel.grid.major=element_blank(),
        panel.grid.minor=element_blank(),
        panel.border=element_blank(),
        axis.line=element_line(),
        legend.position='none', #legend ='right'
        axis.title = element_text(face ="bold"),
        strip.background = element_rect(colour="white", fill="white"))

#remove the grey background on the last facet label that characterizes only the silvoarable summary effect size
arthropods_forest_improved_facet_label <- ggplot_gtable(ggplot_build(arthropods_forest))
stripr <- which(grepl('strip-r', arthropods_forest_improved_facet_label$layout$name))
fills <- c("grey","grey","white")
k <- 1
for (i in stripr) {
j <- which(grepl('rect', arthropods_forest_improved_facet_label$grobs[[i]]$grobs[[1]]$childrenOrder))
arthropods_forest_improved_facet_label$grobs[[i]]$grobs[[1]]$children[[j]]$gp$fill <- fills[k]
k <- k+1
}

#cairo_pdf(file="Figures/Figure_Arthropod_Forestplot.pdf",width =8.1,height =6.75)
grid.draw(arthropods_forest_improved_facet_label)
```

```
#dev.off()
```

# Sensitivity Analysis

## Level-of-evidence weighting

Are the level of evidence and the variance of the effect size correlated?

```
cor.test(ef1$LoEsc,ef1$vi,method="spearman")
```

```
## 
##  Spearman's rank correlation rho
## 
## data:  ef1$LoEsc and ef1$vi
## S = 46940, p-value = 0.2428
## alternative hypothesis: true rho is not equal to 0
## sample estimates:
##       rho 
## 0.1424895
```

Summary effect size and confidence interval based on the level-of-evidence weighting.

```
lme_intercept_LoE <- lme(yi~1,
             random=~1|AuthorSnip,
             weights=varFixed(~vi*LoEsc),
             control=list(sigma=1),
             method="ML",
             data=ef1)

lme_intercept_LoE$coefficients$fixed
```

```
## (Intercept) 
##  0.09328647
```

```
lme_intercept_LoE$coefficients$fixed-1.96*summary(lme_intercept_LoE)[[20]][[2]]
```

```
##  (Intercept) 
## -0.003100533
```

```
lme_intercept_LoE$coefficients$fixed+1.96*summary(lme_intercept_LoE)[[20]][[2]]
```

```
## (Intercept) 
##   0.1896735
```

## Cumulative meta analysis

Cumulative forest plot with ggplot

```
data_ordered <- ef1[order(ef1$Publication_Year),]

#calculate summary effect sizes for each time step
model_cum <- vector("list",nrow(ef1))
for(i in 2:nrow(ef1)){
model_cum[[i]] <- rma.mv(yi, vi, data = data_ordered[1:i,], method = "ML",random = list(~ 1 | AuthorSnip))
}

# arrange data
df_cum <- data.frame(do.call(rbind, model_cum), stringsAsFactors=FALSE)
df_cum <- df_cum %>% select(beta, ci.ub, ci.lb)
df_cum <- df_cum %>% add_row(.before = 1)
df_cum <- cbind(as.data.frame(data_ordered), df_cum)

sel <- apply(df_cum[ , names(df_cum)%in%c("beta","ci.ub","ci.lb")], 2, function(x) as.numeric(as.character(x)))                         
df_cum[ , colnames(df_cum) %in% colnames(sel)] <- sel  

df_cum <- df_cum[order(df_cum$Publication_Year),]
df_cum$label <- paste(nrow(df_cum):1+10) 

# add log response ratio of the first effect size (Hill et al. 1991) from 'ef'
df_cum$beta[df_cum$AuthorSnip == "Hill et al. 1991"] <- ef$yi[ef$AuthorSnip == "Hill et al. 1991"]
df_cum$ci.lb[df_cum$AuthorSnip == "Hill et al. 1991"] <- df_cum$beta[df_cum$AuthorSnip == "Hill et al. 1991"] - ef$vi[ef$AuthorSnip == "Hill et al. 1991"]
df_cum$ci.ub[df_cum$AuthorSnip == "Hill et al. 1991"] <- df_cum$beta[df_cum$AuthorSnip == "Hill et al. 1991"] + ef$vi[ef$AuthorSnip == "Hill et al. 1991"]

df_cum$AuthorSnip <- as.character(df_cum$AuthorSnip)
df_cum$AuthorSnip <- paste('+',as.character(df_cum$AuthorSnip)[nrow(df_cum):1])
df_cum$AuthorSnip[df_cum$AuthorSnip == "+ Hill et al. 1991"] <- "Hill et al. 1991"
df_cum$AuthorSnip <- as.factor(df_cum$AuthorSnip)

# define colours
cols = c(arth.col, bat.col, bird.col, flb.col, plant.col, "grey") 

# cumulative plot
cum_forest_plot <- ggplot(df_cum, aes(y=label, x=beta, xmin=ci.lb, xmax=ci.ub,color=Biodiversity_Group1,fill=Biodiversity_Group1)) + 
 geom_errorbarh(size=0.8,position=position_dodge(width = 0.5), alpha=1,height=0) +
  geom_point(color = 'black')+ #Add data points and color them black
  geom_vline(xintercept=0, color='black', linetype='dashed')+   #Add a vertical dashed line indicating an effect size of zero
  scale_x_continuous(limits=c(-0.25,0.5), name='Log Response Ratio')+ #Specify the limits of the x-axis and relabel it
  scale_y_discrete(label = df_cum$AuthorSnip)+ # numbers refer to row numbers of dat
  ylab('Reference')+ #y-axis label
  scale_fill_manual(values=cols, guide = FALSE)+
  scale_color_manual(values=cols, name = "", labels=c("Arthropods","Bats","Birds", "Fungi, Lichens,\n& Bryophytes", "Plants", "Multiple"))+ # legend title
  theme_bw()+
  theme(panel.grid.major=element_blank(),
        panel.grid.minor=element_blank(),
        panel.border=element_blank(),
        axis.line=element_line(),
        legend.position='right',
        axis.title = element_text(face ="bold"))

cum_forest_plot
```

## Funnel plot

Funnel plot for the mixed-effects model (according to Nakagawa and Santos 2012). The normalized residuals are symmetrically distributed in a funnel plot and suggest no publication bias.

```
#cairo_pdf(file="Figure_Funnel_Plot.pdf",width =11,height =7)
plot(sqrt(ef1$vi)~residuals(m2_lme,type="normalized"),ylim=c(1,-1.8),pch=16,ylab="Standard error",xlab="Normalized residuals") 
rect(par("usr")[1], par("usr")[3], par("usr")[2], par("usr")[4], col ="grey")
lines((seq(1,-3,length.out=50)+1.96),seq(1,-3,length.out=50))
lines((-seq(1,-3,length.out=50)-1.96),seq(1,-3,length.out=50))
polygon(c(rev(-seq(1,-3,length.out=50)-1.96), seq(1,-3,length.out=50)+1.96), c(rev(seq(1,-3,length.out=50)), seq(1,-3,length.out=50)), col = "white", border = NA)
points(sqrt(ef1$vi)~residuals(m2_lme,type="normalized"),pch=16,cex=0.5) #model without evidence weighting looks very similar.
abline(v=0)
```

```
#dev.off()
```

## Egger’s regressions test

```
b1 <- sqrt(ef1$vi)*sqrt(1/(ef1$vi*ef1$LoEsc))
summary(lm(residuals(m2_lme,type="normalized")*sqrt(1/(ef1$vi*ef1$LoEsc))~b1))
```

```
## 
## Call:
## lm(formula = residuals(m2_lme, type = "normalized") * sqrt(1/(ef1$vi * 
##     ef1$LoEsc)) ~ b1)
## 
## Residuals:
##     Min      1Q  Median      3Q     Max 
## -67.478  -1.563   0.000   1.236  64.515 
## 
## Coefficients:
##             Estimate Std. Error t value Pr(>|t|)
## (Intercept)   0.4278    25.7020   0.017    0.987
## b1           -0.7735    46.0726  -0.017    0.987
## 
## Residual standard error: 15.77 on 67 degrees of freedom
## Multiple R-squared:  4.207e-06,  Adjusted R-squared:  -0.01492 
## F-statistic: 0.0002818 on 1 and 67 DF,  p-value: 0.9867
```

Intercept is not significant. There is no evidence for a publication bias.

# References

Duncan, K W. 1979. “Obtaining the overall mean and variance for combined samples.” *Mauri Ora* 7: 139–42.

Martin, Philip A., Adrian C. Newton, Marion Pfeifer, Min Sheng Khoo, and James M. Bullock. 2015. “Impacts of tropical selective logging on carbon storage and tree species richness: A meta-analysis.” *Forest Ecology and Management* 356: 224–33. https://doi.org/10.1016/j.foreco.2015.07.010.

Mupepele, Anne-Christine, Jessica C. Walsh, William J. Sutherland, and Carsten F Dormann. 2016. “An evidence assessment tool for ecosystem services and conservation studies.” *Ecological Applications* 26 (5): 1295–1301. https://doi.org/10.1890/15-0595.

Nakagawa, Shinichi, and Eduardo S A Santos. 2012. “Methodological issues and advances in biological meta-analysis.” *Evolutionary Ecology* 26 (5): 1253–74. https://doi.org/10.1007/s10682-012-9555-5.

Nakagawa, Shinichi, and Holger Schielzeth. 2013. “A general and simple method for obtaining R2 from generalized linear mixed-effects models.” *Methods in Ecology and Evolution* 4 (2): 133–42. https://doi.org/10.1111/j.2041-210x.2012.00261.x.

Walter, and Breckle. 1991. “{Ö}kologie der Erde, Band 1.” In *{Ö}kologie Der Erde, Band 1*, 3rd ed., 23, 24. Stuttgart: Spektrum Akademischer Verlag.
