# Supplementary figures and images for: European agroforestry has no unequivocal effect on biodiversity: a time-cumulative meta-analysis
Source: BMC Ecol Evol. 2021 Oct 23;21:193. doi: 10.1186/s12862-021-01911-9 (PMC8541809; doi:10.1186/s12862-021-01911-9)

Standard error

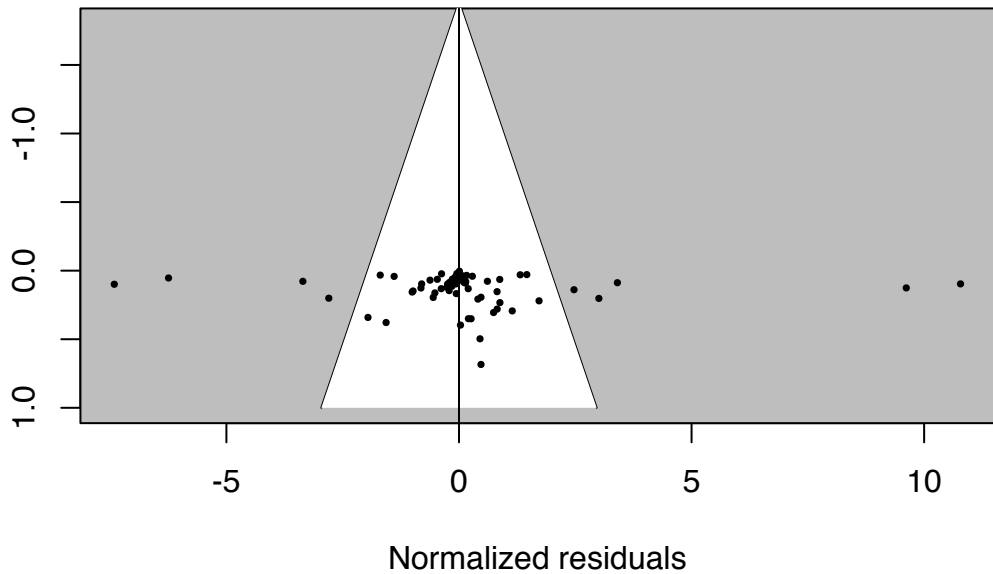

Supplement: Supplementary file 6 — Additional file 6. Funnel Plot. The file is a portal document format (.pdf) with the funnel plot of the analysis. [file 12862_2021_1911_MOESM6_ESM.pdf]
